# ﻿Beyond the species name: an analysis of publication trends and biases in taxonomic descriptions of rainfrogs (Amphibia, Strabomantidae, *Pristimantis*)

**DOI:** 10.3897/zookeys.1134.91348

**Published:** 2022-12-07

**Authors:** Carolina Reyes-Puig, Emilio Mancero

**Affiliations:** 1 Universidad San Francisco de Quito USFQ, Instituto iBIOTROP, Museo de Zoología & Laboratorio de Zoología Terrestre, Quito, 170901, Ecuador; 2 Universidad San Francisco de Quito USFQ, Colegio de Ciencias Biológicas y Ambientales COCIBA, Quito, 170901, Ecuador; 3 Instituto Nacional de Biodiversidad, Unidad de Investigación, Quito, 170506, Ecuador; 4 Biology Department, Grand Valley State University, Allendale, MI 49401, USA

**Keywords:** Author gender, herpetology, inclusion, language bias, new species, taxonomy

## Abstract

The rainfrogs of the genus *Pristimantis* are one of the most diverse groups of vertebrates, with outstanding reproductive modes and strategies driving their success in colonizing new habitats. The rate of *Pristimantis* species discovered annually has increased continuously during the last 50 years, establishing the remarkable diversity found in this genus. In this paper the specifics of publications describing new species in the group are examined, including authorship, author gender, year, language, journal, scientific collections, and other details. Detailed information on the descriptions of 591 species of *Pristimantis* published to date (June 2022) were analyzed and extracted. John D. Lynch and William E. Duellman are the most prolific authors, yet Latin American researchers have scaled up and continued the description processes since the 1990s. The most common language used for descriptions is English, followed by Spanish. The great majority of authors have described only one species. The largest proportion of authors who have participated in the descriptions is of Ecuadorian nationality. Ecuador is the country with the highest description rate per year (3.9% growth rate). Only 20% of the contributions have included women and only 2% have featured women as principal authors. 36.8% of the species described are in the Not Evaluated or Data Deficient categories under the IUCN global red list. The importance of enhancing the descriptions in Spanish is emphasized and the inclusion based on equal access to opportunities for female researchers in *Pristimantis* taxonomy is encouraged. In general, if the current trends in *Pristimantis* descriptions continue, in ten years, a total of 770 or more species described could be expected.

## Abstract (es)

Las ranas de la lluvia del género *Pristimantis* es uno de los grupos de vertebrados más diversos, con una variedad de modos reproductivos y estrategias que impulsan su éxito en la colonización de nuevos hábitats. La tasa de especies de *Pristimantis* descubiertas anualmente ha aumentado continuamente durante los últimos 50 años, estableciendo la notable diversidad encontrada en este género. En este artículo, examinamos los detalles de las publicaciones que describen nuevas especies en el grupo, incluida la autoría, el año, el idioma, la revista, el género, las colecciones científicas y otros detalles. Analizamos y extrajimos información detallada sobre las descripciones de 591 especies de *Pristimantis* publicadas hasta la fecha (junio 2022). John D. Lynch y William E. Duellman son los autores más prolíficos, pero los investigadores latinoamericanos han ampliado y continuado los procesos de descripción desde la década de 1990. El idioma más común utilizado para las descripciones es el inglés, seguido del español. La gran mayoría de los autores han descrito una sola especie. La mayor proporción de autores que han participado en las descripciones es de nacionalidad ecuatoriana. Ecuador es el país con la tasa de descripción más alta por año (tasa de crecimiento del 3,9%). Solo el 20% de las contribuciones han incluido a mujeres y solo el 2% las ha presentado como autoras principales. El 36,8% de las especies descritas se encuentran en las categorías No evaluadas o Datos insuficientes de la lista roja mundial de la UICN. Destacamos la importancia de potenciar las descripciones en español y fomentar la inclusión de mujeres investigadoras en la taxonomía de *Pristimantis*. En general, si continúan las tendencias actuales en las descripciones de *Pristimantis*, en 10 años se podría esperar un total de 770 o más especies descritas.

## ﻿Introduction

*Pristimantis* Jiménez de la Espada, 1870 is a clade of New-World direct-developing frogs belonging to the family Strabomantidae, order Anura, class Amphibia, phylum Chordata. It is the most speciose genus of terrestrial vertebrates with 591 described species to date (Hedges et al. 2008; Frost 2022). A greater focus on molecular, acoustic, and osteological techniques combined with a significant increase in sampling efforts has led to a rise in the number of newly described species in recent years, allowing further research in understanding their taxonomy and systematics (Padial et al. 2010; Hutter and Guayasamin 2015; Kaiser et al. 2015; González-Durán et al. 2017; Reyes-Puig et al. 2020).

The earliest description of this genus was the description of the genus type *Pristimantisgaldi* (Jiménez de la Espada, 1870). The genus was later placed under the synonymy of *Hylodes* sensu lato by Boulenger (1882), then synonymized as *Eleutherodactylus* by Peters (1955). *Cyclocephalus*, *Pseudohyla*, and *Trachyphrynus* were also synonymized with *Eleutherodactylus* by Lynch (1968, 1971). Heinicke et al. (2007) removed *Pristimantis* from the synonymy under *Eleutherodactylus*, with support from molecular evidence. This large and phenotypically diverse genus has faced subsequent molecular analyses confirming its monophyly and status as a closely related taxa to *Lynchius*, *Oreobates*, and *Phyrnopus*, with *Yunganastes* being suggested as well (Pyron and Wiens 2011; Canedo and Haddad 2012; Pinto-Sánchez et al. 2012). Several discernible groups can be found within the genus, originally described based on extensive morphological data and revised on subsequent molecular analyses (Lynch and Duellman 1997; Hedges et al. 2008; Pinto-Sánchez et al. 2012; Padial et al. 2014; Mendoza et al. 2015). There are currently 13 recognized species groups, with *P.conspicillatus* as the largest with 36 species (Padial et al. 2014; González-Durán et al. 2017; Reyes-Puig et al. 2020; Taucce et al. 2020). Several other species remain unassigned as they are demonstrably non-monophyletic. These species can maintain taxonomic value as they can be grouped among phenotypically similar species, thus revealing useful comparative information (Hedges et al. 2008; Padial et al. 2014).

Members of this genus are remarkable for laying eggs in terrestrial habitats, with the embryos developing directly into frogs, bypassing the aquatic stage of their lifecycle (Woolbright 1985; Duellman and Lehr 2009). Assemblages of *Pristimantis* species are common, as their morphology and therefore behavioral and ecological activities are remarkably similar (Arroyo et al. 2008). They are characterized as insectivorous generalists, choosing prey depending on availability and size (Lynch and Duellman 1997; Arroyo et al. 2008). Individuals are predominantly arboreal and nocturnal, commonly perching on leaves at heights below 200 cm. As their reproductive biology leads them away from congregating at ponds, a common strategy for males in this genus is to vocalize from the ground or a suitable perch in search of a mate (Lynch and Duellman 1997; Duellman and Lehr 2009).

The genus is widely distributed throughout the New World and considered the most extensive among Neotropical amphibians, with species found in tropical and subtropical forests in South America and up to lower Central America (Lynch and Duellman 1997; Pinto-Sánchez et al. 2012; Meza-Joya and Torres 2016; Armesto and Señaris 2017). The group shows particularly high levels of diversity and endemism in the Andes, predominantly at elevations above 2000 m along humid environments and surrounding lowlands and Chocoan South America (Heinicke et al. 2007; Meza-Joya and Torres 2016; Armesto and Señaris 2017; Reyes-Puig et al. 2020). The diversity of the genus is greater in Colombia, Ecuador, and Peru and the piedmont, montane, and montane cloud forests of the western and eastern slopes of the three countries concentrate the highest levels of endemism (Hedges et al. 2008; Ron et al. 2022).The continuous increase in the number of species described within this genus suggests a much greater species richness than anticipated, with subtle differences in behavior and physiology reflecting distinct evolutionary pathways (de Queiroz 2005; Hutter and Guayasamin 2015).

The conditions leading to such a high diversification rate in *Pristimantis* are not completely understood. Families of direct-developing frogs diverged quickly during the early to middle Cenozoic, favoring a wide dispersion across a range of habitats in South America, leading to a rapid accumulation of population genetic isolation (Heinicke et al. 2007; Heinicke et al. 2009; Pröhl et al. 2010). An important point of the radiation of the genus is found between 1000 and 3000 m in the northwestern Andes (Mendoza et al. 2015). The complex biogeographic dynamics in the area not only supported allopatric speciation, but also facilitated dispersion of lowland species during the Paleocene and Pliocene, leading to a speciation pattern particularly suitable to originate cryptic and sibling lineages (Lynch and Duellman 1997; Mendoza et al. 2015).

In the last 20 years, the increase in species description rates in South America has shed light on the remarkable diversity and endemism of *Pristimantis*, while hinting at the complex patterns of speciation taking place. Given the cryptic nature found in members of this genus, the work to discover and describe new species appears far from over. Categorizing these frogs and their characteristics in a taxonomic context presents several challenges, further obscured by the high degree of plasticity evidenced across the group (Guayasamin et al. 2015).

Despite the fact that this genus is so diverse and taxonomic and systematic research continues from year to year, information on publication patterns and trends is scarce. Issues such as gender and language biases in publications have not yet been explored. Within the biological sciences, Zoology in particular, studies in this branch have been characterized as male-dominated, imposing limitations in the professional development of many women (Slobodian et al. 2021). Similarly, it has been shown that, for example, in ecology and zoology the proportion of principal investigators publishing with women is lower compared to the proportion with men (Salerno et al. 2019). On the other hand, the language of publication continues to be dominated by the English language, although there have been approaches to improve the transmission of science so that it can be disseminated locally (Ramírez-Castañeda 2020). Latin American researchers not only tend to be under pressure to publish in English, but also to do so with colleagues from developed countries, issues that tend to be related to the number of citations an article can receive (Meneghini et al. 2008). Thus, in this article we aim to delve into the specifics of the biases and trends in publication of descriptions of new species of *Pristimantis* by carrying out a detailed review of all description parameters, emphasizing location, authorship (including author gender), scientific collection, and language.

## ﻿Materials and methods

We followed the proposal of Hedges et al. (2008) and Heinicke et al. (2018) for family classification of *Pristimantis*. The updated list of species formally described to date was extracted from the Amphibian Species of the World web page from the American Museum of Natural History (Frost 2022). On 14 June 2022, the *Pristimantis* list contained 591 described species. Based on this list, we generated a detailed database for each species, extracting specific data available on the original description and the aforementioned web page. We built a database with the following fields: unique species identifier, species, journal, year of publication, first author, authors, gender of the author, nationality of authors, corresponding author, genus, country of description, type locality, holotype, synonymy, species group (if applicable), distribution, language of the description, institutional affiliations, conservation status and type of description. For more details see Suppl. material 1. We calculated the growth rate of global descriptions and that of the seven countries with the highest number of descriptions (i.e., Ecuador, Colombia, Peru, Venezuela, Brazil, Panamá, Bolivia). In order to better represent the different historical trends seen in *Pristimantis* description, growth rates are divided into two periods: The first from 1958–1989, when descriptions begin to be more constant, and the second one from 1990–2021. Due to a significant increase in descriptions at the time, the period ranging from 2010–2021 is also considered. This is mainly because we intend to identify a realistic description rate with the latest advances in the taxonomy and systematics of the genus. In addition to the fact that the number of researchers currently working in *Pristimantis* taxonomy is greater than in previous periods (Suppl. material 1). We did not consider the periods prior to 1958, as there is no significant growth in description rates observed during them.

We carried out the search for publicly available information regarding gender and nationality of authors through individual exploration enabled by the Google search engine. This search was based on exploration of the Google search results associated with the names of authors (as self-reported in publications describing *Pristimantis* species) throughout the web. Priority was given to information associated with institutional affiliation and research endeavors, cross-referenced with professional sites such as ResearchGate, LinkedIn and Google Scholar. Information regarding the gender of authors was assessed based on a binary understanding of gender and assumed based on gender roles traditionally assigned to their names, physical appearance, and self-reported identity, when available. Information regarding conservation status was obtained from the IUCN Red List of Threatened Species (IUCN 2022). After finalizing data input, we cleaned and homogenized the database to prevent typographical errors as well as duplicate and empty cells. In order to describe the increase in women involved in *Pristimantis* descriptions over the years, we used generalized linear models (GLMs). These include a quasi-binomial error distribution, necessary given the nature of the data (i.e., dichotomous proportionality data and overdispersion). We defined years as the dependent variable and the proportion of female authors as the independent variable, in order to identify the relationship between the proportion of women featured in descriptions and time. We made a GLM of the total number of female authors and one specific to each of the five countries with the greatest diversity of *Pristimantis* (Ecuador, Colombia, Peru, Venezuela, and Brazil). The timeframe for this analysis is from 1970 onwards, corresponding to the period in which women begin to be active in describing a new species of *Pristimantis*. All management, cleaning, and analysis of the database were performed in the statistical software R (R Core Team 2022); utilizing the “tidyverse”, “forcats” and “gridExtra” packages.

## ﻿Results

**Figure 1.**
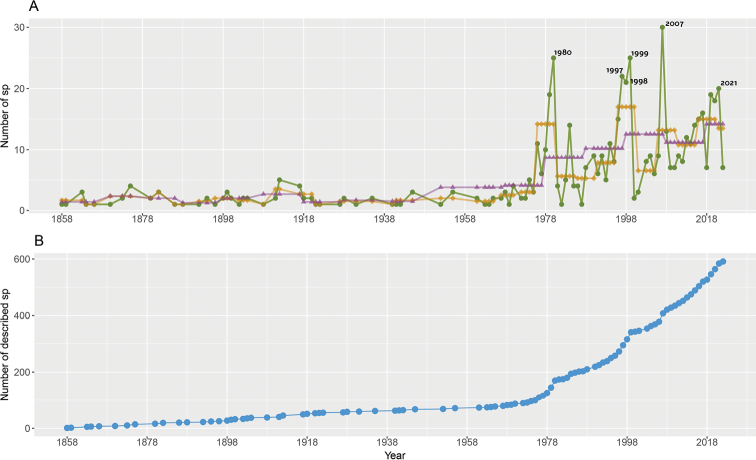
*Pristimantis* descriptions through time **A** species description over 164 years, dots and green lines represent new taxa per year, diamonds and brown lines five-year average, and triangles and purple lines ten-year average **B** cumulative number of species descriptions.

Species descriptions of the group began in 1858 with *Pristimantisconspicillatus* (under the synonymy of *Hylodesconspicillatus*, *Lithodytesconspicillatus*, and later *Eleutherodactylusconspicillatus*). For a century the descriptions of this group remained relatively stable with fewer than four species described on average every ten years (Fig. 1). From the 1960s, the descriptions in the group increased significantly with several peaks towards the 70s but with a particular one towards 1980. The number of descriptions every five years increased in relation to the range between 1958–1970, with an average of 5–14 descriptions between 1978 and 1996. After this year, the most notable peaks in new descriptions were restricted to the years 1997, 1998, 1999, 2007, 2019, 2020, and 2021 (Fig. 1A). The significant increase in the descriptions of new species in this group intensified after 1978 and the accumulation curve of descriptions seems to continue increasing up to the present date, with a total of 591 formally described species (Fig. 1B). The period with the highest annual rates of *Pristimantis* description is found between 1958–1989. For Ecuador, the period with the highest description rate lies between 2010–2021, for Colombia between 1958–1989, for Peru between 1990–2021, and for both Brazil and Bolivia, a peak in the description rate is observed in the period 2010–2021 and 1990–2021, respectively. However, it is important to consider that in order to calculate the growth rate, the initial and final values of the numbers of described species are taken into account. By having such a low number of total descriptions, if the species double from one year to the next, the rate is higher; however, the total number of species is much lower compared to that of other countries (Table 1).

**Table 1.** Growth rates in *Pristimantis* descriptions.

Time series	Total	Ecuador	Colombia	Peru	Venezuela	Brazil	Panama	Bolivia
1958–1989	3.5	4.4	4.1	3.9	3.2	1.4	0	0
1990–2021	3.2	2.6	3.7	5.4	3.7	3.3	2.4	6.1
2010–2021	2.6	4.7	1.02	2.1	2.3	6.1	2.7	0
Total number of descriptions*	591	212	168	107	56	16	9	5

* Number of total descriptions in each country, growth rates instead are calculated as new descriptions - past descriptions / past descriptions.

**Figure 2.**
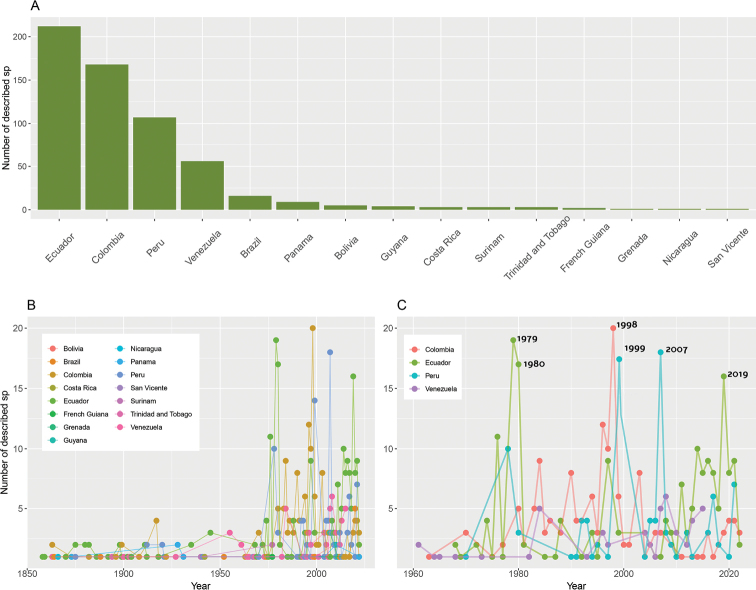
Descriptions of *Pristimantis* species across countries **A** number of species descriptions per country **B** number of annual species descriptions by country **C** number of annual species descriptions in the four countries with the highest species richness.

The distribution of *Pristimantis* extends from Honduras to Peru, specifically, Honduras east through Central America through Colombia and Ecuador to Peru, Bolivia, northern Argentina, and Amazonian and Atlantic Forest Brazil and the Guianas; Trinidad and Tobago; and Grenada, Lesser Antilles (Frost 2022). Ecuador is the country with the highest number of described species with 212 total descriptions, followed by Colombia (168 descriptions), Peru (107 descriptions), and Venezuela (56 descriptions). Other countries within the range of this genus report fewer than ten descriptions (Fig. 2A). From the 1960s onwards, descriptions occurred predominantly in Ecuador, Colombia, and Peru, with at least two peaks of descriptions higher than the average of ten new species described per year (Fig. 2B). Ecuador exhibits three important peaks in species description (i.e., 1979, 1980, and 2019), with more than 15 new species described per year; Colombia peaks in 1998 reaching 20 descriptions in a single year, setting a record in descriptions for this genus; and finally, Peru peaks twice (i.e., 1999 and 2007) reaching 13 and 15 species descriptions, respectively (Fig. 2C).

**Figure 3.**
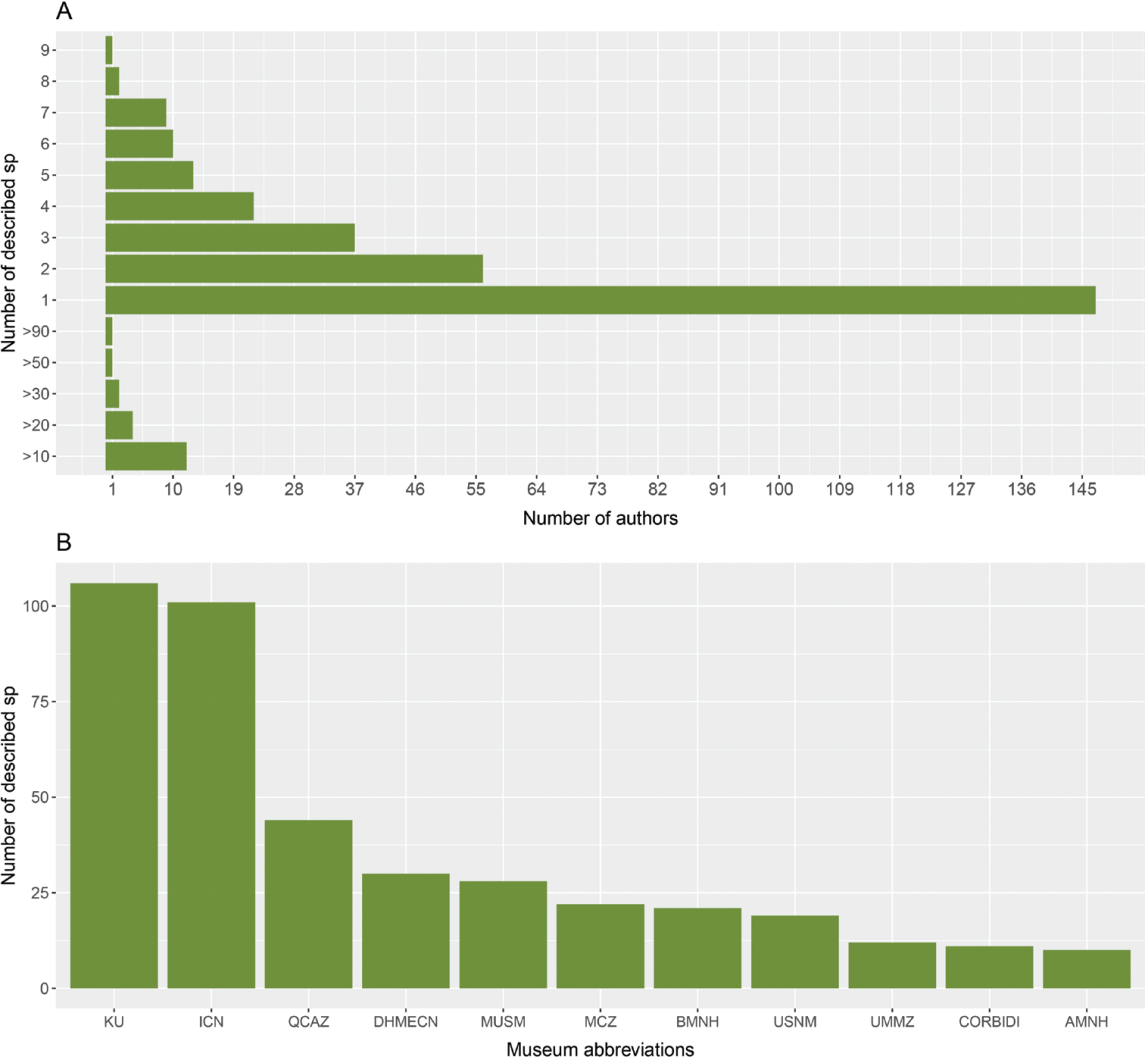
**A** Number of species described by the number of authors **B** number of holotypes of *Pristimantis* housed in the main scientific collections of natural history (an expanded list can be found in Suppl. material 1).

In total, 320 researchers are formally recognized as authors and co-authors in publications describing new species of *Pristimantis* (Suppl. material 1). Almost half (46%) of them have described a single species, while another 46% have described between two and seven species, 6.5% of authors have described 8–30 species, and finally only 0.6% of authors have described more than 50 species (Fig. 3A). Lynch is the author with the highest count of newly described species, with 195 species descriptions to his name. He is followed by Duellman with 82, and E. Lehr and M. Yánez-Muñoz with 36 each. Details on the 20 authors with the highest count of newly described species are provided in Table 2. Across all scientific collections where the type material has been deposited, the four institutions with the highest number of holotypes are the Museum of Natural History of University of Kansas (KU), Museo de Historia Natural del Instituto de Ciencias Naturales de Universidad Nacional de Colombia (ICN), Museo de Zoología de la Pontificia Universidad Católica del Ecuador (QCAZ), and División de Herpetología, Instituto Nacional de Biodiversidad, former Museo Ecuatoriano de Ciencias Naturales (DHMECN). KU and ICN house 35% of the typical material, and QCAZ and DHMECN have the 12.5% of type specimens of the genus (Fig. 3B). The rest of the collections hold a lower proportion of specimens than those mentioned (Fig. 3B, Suppl. material 1).

**Table 2.** Top 100 authors ranked by the number of *Pristimantis* species they have described and country of origin. Authors with three or more species descriptions are included.

Author rank	Author (country)	No of described species	Author rank	Author (country)	No of described species
**1**	**J. D. Lynch (USA)**	**195**	51	D. Szekely (Romania)	5
**2**	**W. E. Duellman (USA)**	**82**	52	D. Zumel (Ecuador)	5
**3**	**E. Lehr (Germany)**	**36**	53	E. A. de Oliveira (Brazil)	5
**4**	**M. H. Yánez-Muñoz (Ecuador)**	**36**	54	E. J. Hernandez-Ruz (Brazil)	5
**5**	**S. R. Ron (Ecuador)**	**30**	55	G. A. Rivas-Fuenmayor (Venezuela)	5
**6**	**P. M. Ruiz-Carranza (Colombia)**	**25**	56	M. B. Perez (Ecuador)	5
**7**	**G. A. Boulenger (Belgium, Great Britain)**	**23**	57	T. Barbour (USA)	5
**8**	**J. M. Guayasamin (Ecuador)**	**20**	58	W. C. H. Peters (USA)	5
**9**	**C. Reyes-Puig (Ecuador)**	**18**	59	L. R. Rodrigues (Brazil)	4
**10**	**C. L. Barrio-Amoros (Spain)**	**17**	60	C. J. Goin (USA)	4
**11**	**J. B. Pramuk (USA)**	**17**	61	D. Armijos-Ojeda (Ecaudor)	4
**12**	**J. V. Rueda-Almonacid (Colombia)**	**16**	62	D. B. Means (USA)	4
**13**	**J. P. Reyes-Puig (Ecuador)**	**15**	63	D. Buckley (Spain)	4
**14**	**M. C. Ardila-Robayo (Colombia)**	**15**	64	D. M. Cochran (USA)	4
**15**	**J. A. Rivero (Puerto Rico)**	**13**	65	D. Rödder (Germany)	4
**16**	**D. Batallas (Ecuador)**	**12**	66	E. R. Wild (USA)	4
**17**	**A. Catenazzi (USA)**	**11**	67	E.A. Pereira (Brazil)	4
**18**	**E. La Marca (Venezuela)**	**11**	68	J. C. Cusi (Peru)	4
**19**	**J. Brito-Molina (Ecuador)**	**11**	69	J. Culebras (Spain)	4
**20**	**N. B. Paez (Ecuador)**	**11**	70	J. G. Martinez (Colombia)	4
21	D. F. Cisneros-Heredia (Ecuador)	10	71	J. J. Mueses-Cisneros (Colombia)	4
22	J. C. Sánchez-Nivicela (Ecuador)	10	72	J. M. Padial (Spain)	4
23	S. B. Hedges (USA)	10	73	J. M. Savage (USA)	4
24	R. von May (USA)	9	74	K. L. A. Guimarães (Brazil)	4
25	M. Rivera-Correa (Colombia)	8	75	L. Alves da Silva (Brazil)	4
26	P. A. Burrowes (USA)	8	76	M. J. Navarrete (Ecuador)	4
27	A. F. Arteaga-Navarro (Ecuador)	7	77	M. Jimenez de la Espada (Spain)	4
28	C. Aguilar (Peru)	7	78	M. Penhacek (Brazil)	4
29	F. J. M. Rojas-Runjaic (Venezuela)	7	79	P. J. R. Kok (Belgium)	4
30	G. Flores (USA)	7	80	S. Duarte-Marín (Colombia)	4
31	H. M. Ortega-Andrade (Ecuador)	7	81	A. Almendariz (Ecuador)	3
32	J. H. Valencia (Ecuador)	7	82	A. Espinosa de los Monteros (Mexico)	3
33	J. Moravec (Czech Republic)	7	83	A. J. Crawford (USA)	3
34	P. J. Venegas (Peru)	7	84	A. M. Suarez-Mayorga (Colombia)	3
35	V. L. Urgiles (Ecuador)	7	85	A. Varela-Jaramillo (Ecuador)	3
36	C. W. Myers (USA)	6	86	C. F. Walker (USA)	3
37	H. Kaiser (USA)	6	87	C. Teran (Ecuador)	3
38	J. A. Ortega (Ecuador)	6	88	E. E. Infante-Rivero ()	3
39	J. C. Chaparro (Peru)	6	89	E. R. Dunn (USA)	3
40	J. J. Ospina-Sarria (Colombia)	6	90	F. H. Test (USA)	3
41	J. M. Daza (Colombia)	6	91	F. Werner (Austria)	3
42	M. A. Donnelly (USA)	6	92	G. A. González-Durán (Colombia)	3
43	M. Schmid (Germany)	6	93	G. A. Maldonado-Castro (Ecuador)	3
44	P. Szekely (Romania)	6	94	J. C. Jordan (Peru)	3
45	S. M. Ramirez-Jaramillo (Ecuador)	6	95	J. Carrion (Ecuador)	3
46	A. G. Ruthven (USA)	5	96	J. J. Morrone (Argentina-Mexico)	3
47	C. R. Hutter (USA)	5	97	J. Köhler (Germany)	3
48	C. Steinlein (Germany)	5	98	J. Lescure (France)	3
49	D. C. Cannatella (USA)	5	99	J. S. Eguiguren (Ecuador)	3
50	D. J. Santana (Brazil)	5	100	K. Sui-Ting (Peru)	3

**Figure 4.**
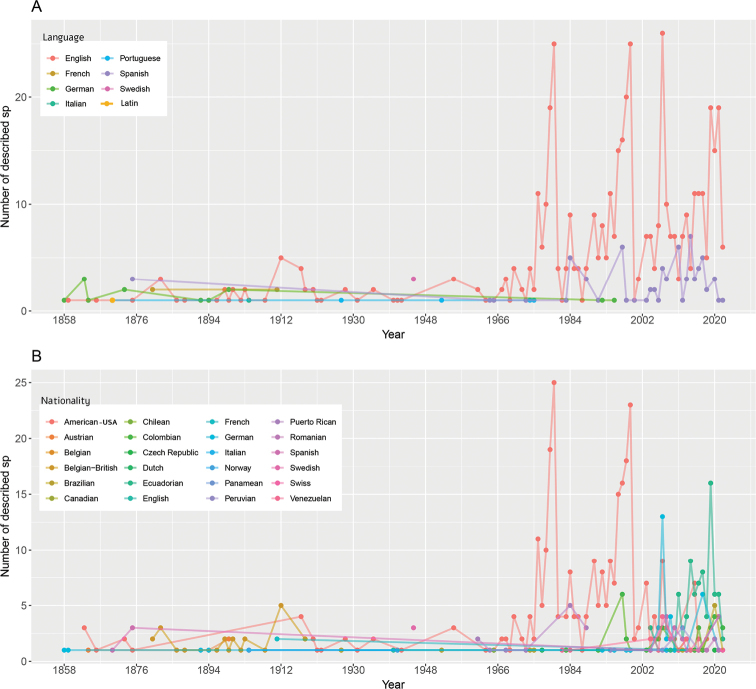
Languages and nationalities of authors who have participated in the descriptions of *Pristimantis* species **A** main languages used in descriptions per year **B** main nationalities of authors involved in descriptions per year.

We identified a variety of eight languages used for the description of new *Pristimantis* species (Fig. 4A, Table 3), with the most common ones being English and Spanish. The English language has dominated species descriptions since the 1960s. The number of descriptions published in Spanish started to increase during the 1980s, though it remains lower than those published in English (Fig. 4A, Table 3). Conversely, there are more than 20 nationalities of researchers who have participated as authors and co-authors in descriptions of new species of *Pristimantis* (Fig. 4B). Researchers from the USA have significantly dominated, not only the total number of descriptions, but also the number of annual descriptions until 2002. As of this year, the participation of Ecuadorian authors has increased in greater numbers compared to their Colombian, Peruvian, and Venezuelan peers (Fig. 4B). The greatest percentage of authors involved in descriptions of new *Pristimantis* species are of Ecuadorian nationality (20.9%), followed by US Americans (18.8%) and Colombians (15.3%) (Table 4).

**Table 3.** The most common languages in which new species of *Pristimantis* have been described.

Language	Number of descriptions	Percentage of taxa
English	492	83.2
Spanish	73	12.4
German	13	2.2
Portuguese	4	0.7
French	4	0.7
Swedish	3	0.5
Italian	1	0.2
Latin	1	0.2

**Table 4.** The most common author nationalities in *Pristimantis* descriptions.

Nationalities	Number of authors	Percentage of authors
Ecuadorian	67	20.9
US Americans	60	18.8
Colombian	49	15.3
Brazilian	30	9.4
Peruvian	21	6.5
German	18	5.6
Panamanian	7	2.2

**Figure 5.**
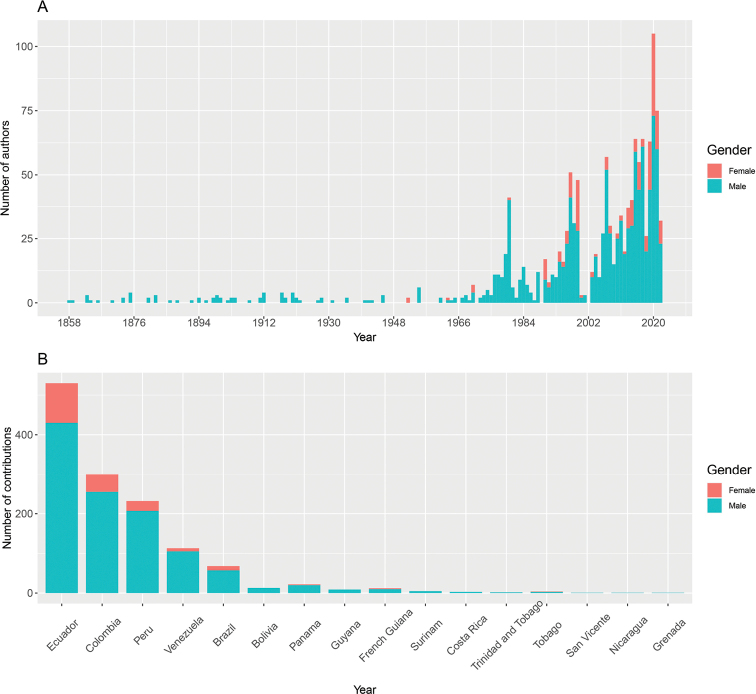
Male and female contributions to *Pristimantis* descriptions **A** number of female and male authors that participated in descriptions per year **B** contributions of female and male authors to descriptions per country.

**Figure 6.**
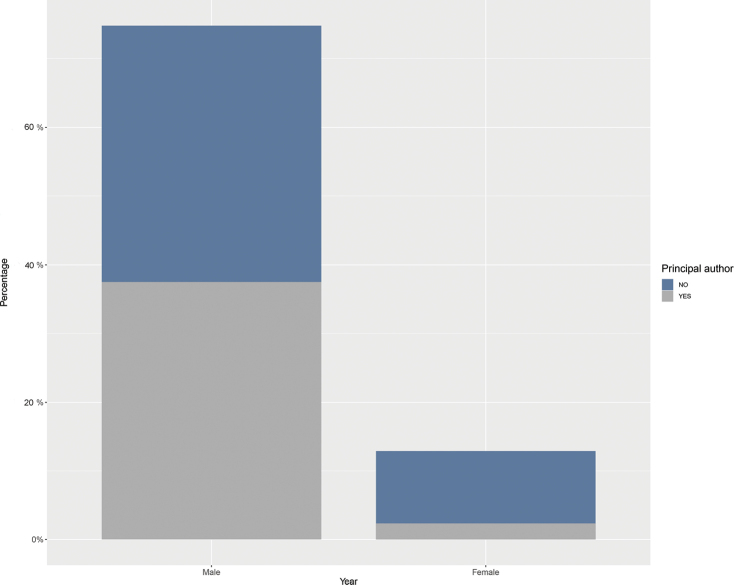
Contributions in which authors have been principal author and defined as either first or corresponding author.

**Figure 7.**
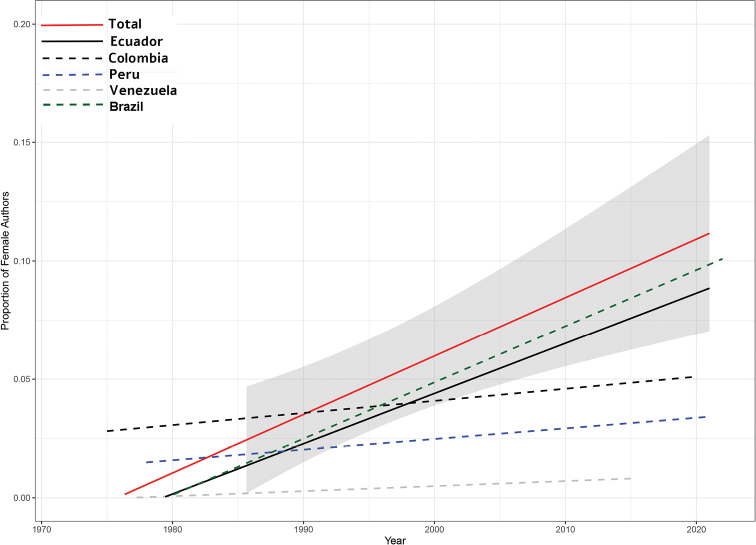
Change in the proportion of female authors over time. Gray shadow represents the confidence intervals of the Generalized Linear Model of the total proportion of female researchers (red solid line). Solid lines depict significant GLMs.

From a gender perspective, 80% of the authors who have participated in the descriptions are male researchers and 20% female researchers (Figs 5, 6). In spite of an increase in the number of descriptions involving women starting in the 1950s, the participation of women in the description process continues to be considerably lower than that of their male counterparts (Fig. 5A). Ecuador is the country with the highest percentage of descriptions that incorporate female researchers with almost 18.8%, while Colombia, Peru, Venezuela, and Brazil have lower percentages (Fig. 5B). The bias towards male authors is overwhelmingly disproportionate in terms of principal authorship (i.e., first author or corresponding author). Where 50% of male authors featured as main authors, only 2% of female authors have held this role (Fig. 6, Table 5). We detected a significant slope between the proportion of female authors who participated in descriptions of all *Pristimantis* and the years (estimate = 0.03, SE = 0.01, *t* = 2.2, *p* = 0.02; Fig. 7). Of the four countries with the highest number of *Pristimantis* species descriptions, Ecuador was the only one with a significant positive slope between the proportion of female authors and time (estimate = 0.09, SE = 0.02, *t* = 3.8, *p* = < 0.001), while that on the contrary Colombia (estimate = 3.7 *10-3, SE = 0.01, *t* = 0.2, *p* = 0.8), Peru (estimate = -0.01, SE = 0.02, *t* = 3.8, *p* = 0.5),Venezuela (estimate = -0.01, SE = 0.03, *t* = -0.5, *p* = 0.7), and Brazil (estimate = -0.04, SE = 0.02, *t* = -2.25, *p* = 0.06) did not show any correlation between these variables (Fig. 7).

**Table 5.** Top 21 female authors ranked by the number of species they have described. * Principal author is defined as being either the first or corresponding author.

#	Author	Nationality	Number of taxa	Principal author*
1	C. Reyes-Puig	Ecuadorian	18	13
2	J. B. Pramuk	US American	17	17
3	M. C. Ardila-Robayo	Colombian	15	-
4	N. B. Paez	Ecuadorian	11	11
5	P. A. Burrowes	US American	8	-
6	V. L. Urgiles	Ecuadorian	7	5
7	J. A. Ortega	Ecuadorian	6	-
8	M. A. Donnelly	US American	6	-
9	D. Szekely	Romanian	5	-
10	M. B. Perez	Ecuadorian	5	-
11	M. J. Navarrete	Ecuadorian	5	2
12	D. M. Cochran	US American	4	3
13	K. L. A. Guimarães	Brazilian	4	-
14	A. Almendariz	Ecuadorian	3	-
15	A. M. Suarez-Mayorga	Colombian	3	-
16	A. Varela-Jaramillo	Ecuadorian	3	-
17	C. Teran	Ecuadorian	3	-
18	G. A. Maldonado-Castro	Ecuadorian	3	-
19	K. Sui-Ting	Peruvian	3	-
20	P. Bejarano-Muñoz	Ecuadorian	3	-
21	Y. Sagredo	Ecuadorian	3	-

**Figure 8.**
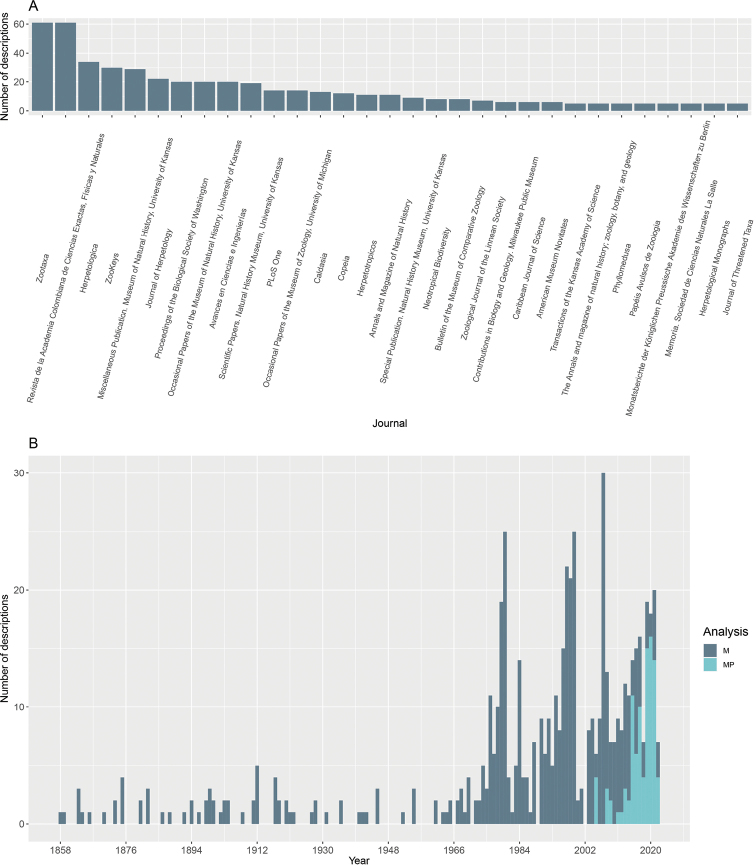
**A** Main scientific journals where descriptions of new *Pristimantis* species are published **B** type of descriptions per year, M = Morphological description, MP = morphological and phylogenetic description.

In relation to peer-reviewed journals in which the descriptions of new species of the genus have been published, Zootaxa and the Revista de la Academia Colombiana de Ciencias, Exactas, Físicas y Naturales have published the highest number of descriptions with 20.6% of the total described species. Other journals such as Herpetologica, ZooKeys, and Miscellaneous Publication of Museum of Natural History of University of Kansas have published 15.7% of the descriptions of *Pristimantis* (Fig. 8A). From the beginning of the descriptions of this diverse genus until the first decade of the 2000s, descriptions have been based mainly on morphology. Phylogenetic analyses were gradually incorporated into descriptions over the last 12 years, leading to a significant reduction in morphology-only descriptions of new *Pristimantis* species (Fig. 8B).

**Figure 9.**
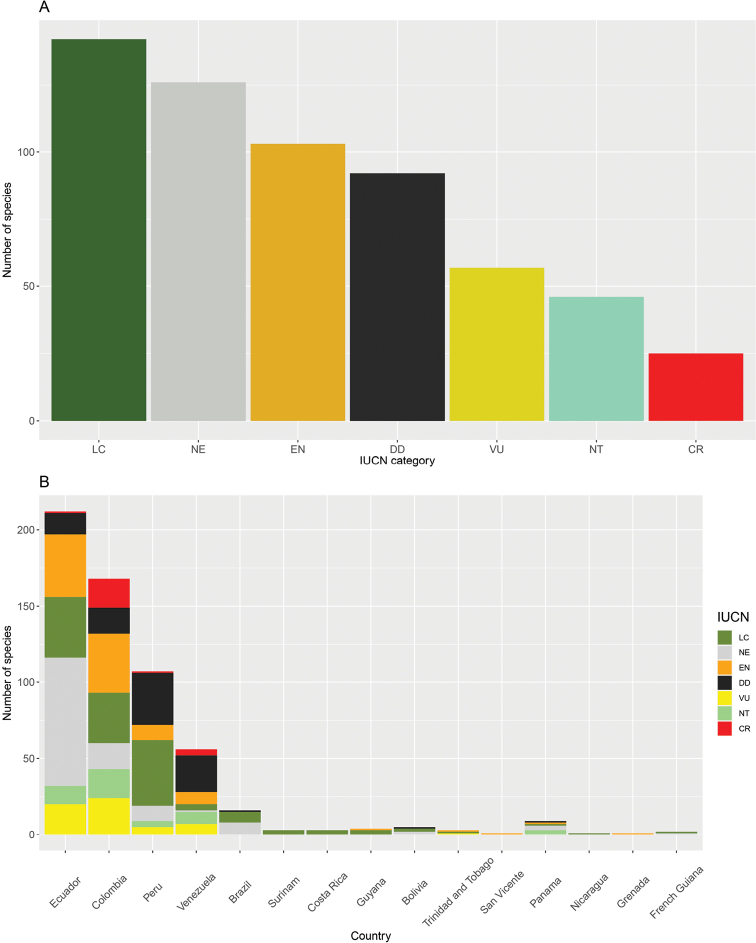
Conservation status categories established by the IUCN Red List for *Pristimantis* species **A** number of species per category **B** number of species in each category per country.

Regarding the conservation status of *Pristimantis* species, 24% are categorized by the IUCN Reed List as Least Concern, 31% are threatened (i.e., CR, EN, or VU), and 36.8% are Not Evaluated or Data Deficient (Fig. 9A). The country with the highest number of Not evaluated species is Ecuador (i.e., 39% of all Ecuadorian species). Peru and Venezuela host the highest percentages of species under the Data Deficient category, 31.8% and 42.8% of their total species, respectively. In Ecuador and Colombia, at least 30% of *Pristimantis* species are under some form of threat (Fig. 9B).

## ﻿Discussion

### ﻿Most prolific authors and countries with the highest numbers of descriptions

The contributions made by Lynch and Duellman to the advancement of *Pristimantis* taxonomy and systematics since the 70s are indisputable. Their most significant contributions focus on large compendiums that include analyses of distribution patterns and advances in systematics and descriptions of several new species (Lynch 1979; Lynch and Duellman 1980, 1997; Duellman and Pramuk 1999). Their work initially focused on the eastern slopes of Ecuador (Lynch 1979; Lynch and Duellman 1980) and later towards the 1990s their interests shifted over to the Colombian and Peruvian foothills (Lynch 1998; Duellman and Pramuk 1999). The most representative works and the most productive years for the descriptions of new species of this genus were:

- 1979 with “Leptodactylid frogs of the genus *Eleutherodactylus* from the Andes of southern Ecuador” by J. D. Lynch and the description of 16 new species;
- 1980 with “ *Eleutherodactylus* of the Amazonian slopes of the Ecuadorian Andes (Anura: Leptodactylidae)” by Lynch and Duellman, which includes 12 new species;
- 1997 with “Frogs of the genus *Eleutherodactylus* in western Ecuador” by Lynch and Duellman;
- 1998 with “New frogs of the genus *Eleutherodactylus* from the eastern flank of the northern Cordillera Central of Colombia” by Lynch and Rueda-Almonacid and “New species of *Eleutherodactylus* from the Cordillera Occidental of western Colombia with a synopsis of the distributions of species in western Colombia” by Lynch, in which nine new species are described;
- 1999 with “Frogs of the genus *Eleutherodactylus* (Anura: Leptodactylidae) in the Andes of northern Peru” by Duellman and Pramuk, describing more than 15 species;
- 2007 with “Three new species of *Pristimantis* (Anura: Leptodactylidae) from the Cordillera de Huancabamba in northern Peru” and “New eleutherodactyline frogs (Leptodactylidae: *Pristimantis*, *Phrynopus*) from Peru” both by Lehr with three and four new species each;
- 2019 with “Systematics of *Huicundomantis*, a new subgenus of *Pristimantis* (Anura, Strabomantidae) with extraordinary cryptic diversity and eleven new species” by Páez and Ron which added 11 new species to Ecuador after almost 40 years of a contribution that includes joint descriptions of *Pristimantis*, as previously published by Lynch and Duellman (Lynch 1979; Lynch and Duellman 1980);
- 2021 with several descriptions from different authors (see Suppl. material 1). We assume that the pandemic due to COVID 19 had a negative effect on field trips in all countries actively working on *Pristimantis* taxonomy. In addition, pure research activities were probably reduced by the effect of the lockdown and COVID 19, as has been observed in other lines of research (Donthu and Gustafsson 2020).

If current trends in the growth rate of annual *Pristimantis* descriptions are maintained and taking into consideration the entire temporal history of descriptions of each country, the total number of species of is expected to increase in the next 10 years to ~777 described species, with ~299 in Ecuador, ~217 in Colombia and ~153 in Peru, ~73 in Venezuela, ~22 in Brazil, ~11 in Panama, and ~6 in Bolivia.

#### Why does Ecuador lead in the number of new species of *Pristimantis* described?

The contributions of Lynch and Duellman in defining the group as a diverse and highly endemic genus became extremely influential to local researchers, leading to further discoveries mainly around Ecuador, Colombia, and Peru (Lynch 1979; Lynch and Duellman 1980, 1997). Despite being the smallest by area of these three, Ecuador sports both the highest number of descriptions of new species of *Pristimantis* and the greatest richness of the genus, followed by Colombia and Peru, suggesting that the known diversity of the genus is underestimated in these countries (Frost 2022; Ron et al. 2022). Out of the six peaks Pistimantis described per year, three of them took place in Ecuador, highlighting the commitment to taxonomic efforts in the region. In addition, Ecuador has had more academic approach to amphibian taxonomy more noticeable than it's neighboring countries: some Ecuadorian taxonomists often pursue higher education abroad in the USA and return to the country to develop their lines of research (e.g., Santiago R. Ron, Juan Manuel Guayasamin, Luis Coloma). However, younger generations of ecuadorian taxonomists have contributed significantly to the taxonomy and systematics of the group, despite not being trained abroad (e.g., Mario H. Yánez-Muñoz, Carolina Reyes-Puig, Juan P. Reyes-Puig, Jorge Brito Molina, Diego Batallas) (Table 2). However, it is important to consider that alliances with colleagues, researchers, and institutions have been crucial for the advancement of *Pristimantis* descriptions in Ecuador. As mentioned by Costello et al. (2013) it seems that South America would have an increase in the number of taxonomists in general, but this is related to the region being much more diverse than others. The Convention on Biological Diversity has proposed some strategies to improve the productivity of taxonomy, including collaborations with both national and international researchers, the requirement to include national institutions when the research is being carried out by foreign researchers, etc. Ecuador has been successful in this context, and this is evidenced by its productivity.

John D. Lynch, an US American herpetologist and taxonomist credited with the greatest number of described species; a total of 149. After working 30 years at the University of Nebraska-Lincoln, he became associate professor and curator of herpetology at the Instituto de Ciencias Naturales de la Universidad Nacional de Colombia in 1997. William E. Duellman, a prominent US American zoologist who was Curator Emeritus of the Herpetology Division of the Natural History Museum of University of Kansas (Coloma and Guayasamin 2022), is the second most productive taxonomist with 82 formally described species. Next in the ranking are authors such as Edgar Lehr, M. H. Yánez-Muñoz, and Santiago R. Ron taking up the mantle from Lynch and Duellman, the three of them are responsible for 15% of the total number of descriptions in the genus. Edgar Lehr is a herpetologist at the University of Illinois and his work has focused on amphibian systematics, mainly from Peru (Illinois Wesleyan University 2022). Mario H. Yánez-Muñoz of Instituto Nacional de Biodiversidad and Santiago R. Ron, professor and curator of the Museum of Zoology QCAZ (Museo de Zoología de la Pontificia Universidad Católica del Ecuador), have focused on the taxonomy and systematics of the genus in the Andean slopes and lowlands of Ecuador for the last 15 years (Ron et al. 2020; INABIO 2022).

The first two positions of the most prolific taxonomists for *Pristimantis* are occupied by US American researchers, who account for 46.8% of the known diversity of the genus. However, among the 20 authors with the most descriptions, 70% are Latin American authors following in the footsteps of Lynch and Duellman. The increase of Latin American researchers interested in the genus arises from the empowerment of local science and biodiversity, driving further interest as research goals are met. Although the last few years have seen an increase in the number of researchers interested in describing new species of *Pristimantis*, most of them describe between one and four species, which reflects the number of authors participating in the publications (Suppl. material 1; e.g., Guayasamin et al. 2017).

### ﻿Species description language

English and Spanish are the dominant languages for descriptions of *Pristimantis* species. From a temporal perspective, all the work developed mainly by Lynch and Duellman (Lynch and Duellman 1980, 1997; Duellman and Pramuk 1999; Duellman and Lehr 2009) correlates with language, scientific collections, years of production, etc. Although the number of descriptions published in Spanish has steadily increased since 1980, publication trends have encouraged an increase in English descriptions as well during the last two decades. The irony of this relationship is that most of the current researchers of the genus are Spanish-speaking Latin Americans, publishing in English mainly for other Spanish speakers. Even this article is an example of this conundrum. However, our aim is to visualize the specifics of descriptions in *Pristimantis*, highlighting patterns and trends in order to visualize to a wider audience the information that lies behind a new description in such a diverse group. In addition, as suggested by Ramírez-Castañeda (2020), we included a full Spanish translation of this manuscript as Suppl. material 2.

The publication of results in English is directly related to pressures from academic institutions to publish in high-impact journals, where English is established as the official language of publication. Publishing in this language for non-English speakers can be time-consuming, demanding, and stressful, and some efforts have been made to understand this in the framework of Latin American researchers (Ramírez-Castañeda 2020). Despite English being the common language for communicating science, the incorporation of Spanish for taxonomic groups geographically distributed in Latin American countries could also be a valuable alternative for disseminating results. Therefore, mechanisms to promote the advancement of research in *Pristimantis* published in Spanish in specialized journals (taxonomy and systematics journals) (e.g., Fig. 7) are necessary.

### ﻿Author nationalities

The number of Ecuadorian researchers is proportionally higher than that of other nationalities describing new *Pristimantis* species during the last few years (e.g., Yánez-Muñoz et al. 2010; Guayasamin et al. 2017; Páez and Ron 2019; Reyes-Puig et al. 2020; Ron et al. 2020). However, it is also worth mentioning that the number of authors per description is higher compared to descriptions from the 1980s (e.g., Lynch and Duellman 1980). There are currently descriptions with up to nine authors (e.g., Ron et al. 2020; see Suppl. material 1), and the average number of authors per species described in the 1980s is between 1 and 2, while during the last decade the average number of authors participating in descriptions is between 5 and 6. In many of these multi-author descriptions, most individual authors have very low description rates while the lead author is an experienced researcher in the study of taxonomy and systematics of the genus.

In this paper we also identify a gap in the presence of *Pristimantis* taxonomists in Peru, Venezuela, Brazil, Panama, and Bolivia compared to Ecuador and Colombia. The inclusion and empowerment of local scientists from these countries would seem necessary in order to increase the study of rainfrog species in their territory, which has been mainly led by foreign authors (e.g., Duellman and Pramuk 1999; Catenazzi and Lehr 2018; Lehr et al. 2021) (details on authors, years, and descriptions can be downloaded from Suppl. material 1).

### ﻿Natural history museum collections

Due to the extensive work of Lynch and Duellman, the museums related to their research currently hold the largest amount of type material from *Pristimantis* (i.e., KU and ICN). The QCAZ is an institution with more than 40 years dedicated to safeguarding and researching Ecuadorian biodiversity and has positioned itself as an international benchmark regarding management and open access to scientific collections, not only for amphibians but also for other vertebrates. This institution is notable for hosting holotypes for 44 species of *Pristimantis* until June 2022 (Ron et al. 2022; Torres-Carvajal et al. 2022). Other South American institutions that preserve a large number of holotypes are the DHMECN (División de Herpetología del Instituto Nacional de Biodiversidad, Ecuador) and the MUSM (Museo de Historia Natural, Universidad Nacional Mayor de San Marcos). The DHMECN of Instituto Nacional de Biodiversidad has focused on the taxonomy and systematics of Ecuadorian herpetofauna for the last 15 years, primarily on *Pristimantis* from the Andean slopes of Ecuador (INABIO 2022). The Herpetology Division of MUSM has a long record of researching Peruvian herpetofauna since 1946. However, since 2007 its scientific production related to descriptions of new species has decreased (MUSM 2022). In addition, virtual access to its collections and databases is limited compared to that of Ecuadorian collections (Ron et al. 2022).

### ﻿Author gender

Historically, zoology has been a male-dominated field. The barriers that limited the number of women in scientific fields before the 20^th^ century has led to the study of animals being an exclusively male discipline (Slobodian et al. 2021). Herpetology is no exception, although there are now far more women in the field than men (Rock et al. 2021). In general, the proportion of female authors is lower, at least in the actively publishing population (Salerno et al. 2019). In the case of descriptions of new species of *Pristimantis*, the number of women actively working in this group is low (20% of the authors are women). Consequently, women are poorly featured as principal authors, a position considered to be the most important indicator of the author's role in the description process. Of a total of 66 female authors, only six have been principal authors. It is necessary to reflect on this pattern, as it is consistent with other studies on the underrepresentation of women in science (West et al. 2013; Fox et al. 2018; Salerno et al. 2019). If current trends continue, female participation in *Pristimantis* descriptions would increase to 50% over the next 25 years (2047). This would mean that in this period the proportion of women in the field could potentially be the same as men, considering only authorship and not principal authorship. Ecuador, being the country with the most researchers, also has the largest number of female researchers; however, many of them are thesis students of principal investigators, so their incursion into the study of the taxonomy of the group is temporary. In this article we encourage the active inclusion of female researchers interested in the field of taxonomy, promoting the advancement of the diversity of *Pristimantis*. We also strongly encourage male researchers to open the doors of their laboratories to women, where their roles are not limited to field assistants or specific sections of descriptions, but also as leaders taking on critical positions in particular taxonomic works. The best way to promote this in the future will be through increasing the proportion of women in senior author positions (Salerno et al. 2019).

### ﻿Peer-reviewed journals

The journals with the highest number of contributions of rainfrog descriptions are Zootaxa and Revista de la Academia Colombiana de Ciencias, Exactas Físicas y Naturales. We can identify two critical issues: the first is the problem of taxonomy today (i.e., underestimation of this area of knowledge being considered a basic science), and the second is how to get other journals to climb to better academic positions if they are local, free, and open access. This question does not have a simple answer since the scaling of these journals will depend entirely on metrics such as the impact factor (IF), an index that assesses the relative importance of a scientific journal in a particular field. The effect of the IF has repercussions on the endless cycle of not citing journals that do not have a medium high IF. Publishing purely taxonomic articles is becoming increasingly complex, both because of the decrease in the number of researchers interested in the field and the number of journals interested in this topic (Wägele et al. 2011). In 2020, Zootaxa was excluded from the Journal Citation Report (JCR) for 2019 (2020 release) by Clarivate Analytics due to excessive use of self-citations. Following a petition that included more than 3900 signatures from research biologists, Clarivate Analytics reversed the decision (Parise Pinto et al. 2021). This misunderstanding and lack of knowledge on how taxonomy works raises concerns about the difficulty of publishing new species discoveries and which journals can be chosen for such a task. Zootaxa shows its importance not only because it has included descriptions of more than 25% of the world’s known biodiversity (Parise Pinto et al. 2021), but in the case of *Pristimantis* it is the main journal in which descriptions have been published.

### ﻿Description type

It is important to note that in the past the vast majority of new species descriptions have been based on morphologic data (83.7% of total descriptions). However, during the last 20 years the molecular revolution has transformed the description process, including DNA sequences that allow phylogenetic positioning of taxa. The continuous increase in available molecular techniques has made the costs to implement them lower and thus more accessible. Therefore, the inclusion of sequencing has become a common tool to better understand the ancestry-descent relationships of living organisms (Malakhov 2013). However, it is worth considering that in the description of new species, sequencing should not replace a detailed taxonomic assessment with morphological characters that allow observers to clearly differentiate one species from another (Guerra-García et al. 2008). Positioning taxonomy as a necessary science to discover and describe biodiversity is essential in its own right. This is evident in the case of *Pristimantis*, as supported by this review, given the trend of increasing numbers of described species and descriptions of highly cryptic species (Padial and De la Riva 2009; Hutter and Guayasamin 2015; Páez and Ron 2019; Ortega-Andrade et al. 2015).

### ﻿Global threat categories

Finally, regarding the IUCN global conservation status of *Pristimantis*, we highlight that a representative proportion of the species described to date (i.e., 36.8%) are found in uncertainty categories such as Not Evaluated and Data Deficient. These categories reflect the need for a global evaluation of the species of this genus, on account of its high endemism and species richness. On the other hand, it also highlights the increasing rate of species descriptions. Conservation status assessments are generally carried out by the global IUCN every four to five years (IUCN 2022), a period in which there are likely to be 10–15 new species of *Pristimantis* per year. Therefore, global red list assessments will always be one step behind the evaluation of threat criteria. An alternative to address this matter could be through local and regional red list assessments, which would be more readily updated and may include other variables for categorization (Ortega-Andrade et al. 2021). In addition, many of the new species of *Pristimantis* have restricted distribution ranges with populations facing various threats (e.g., Brito-Zapata et al. 2021), implying a higher degree of vulnerability. Nevertheless, if global assessments are not conducted for many of these species, the conservation priorities of the genus remain underestimated. In this work we include the categories proposed by the IUCN global red list (IUCN 2022), since the local and regional evaluations of each country are not updated and standardized and therefore cannot be compared. Ecuador has made the most recent effort for a more complete and up-to-date cataloguing, establishing 50% of Ecuadorian *Pristimantis* species under some degree of threat (Ortega-Andrade et al. 2021). This estimate is considerably higher than that reported by the IUCN of 33.5%, likely due to the proportion of species not evaluated or with insufficient information. All these aspects reinforce the importance of continuing to invest not only in the advancement of taxonomic research on the genus, but also in conservation strategies articulated between countries in the region.

## Supplementary materials

Supplementary material 1 Complete database of *Pristimantis* descriptions Data type database (excel document) Explanation note We present the complete database of information on *Pristimantis* descriptions, including year, authors, nationalities of authors, languages, journals, etc. https://binary.pensoft.net/file/779039This dataset is made available under the Open Database License (http://opendatacommons.org/licenses/odbl/1.0/). The Open Database License (ODbL) is a license agreement intended to allow users to freely share, modify, and use this Dataset while maintaining this same freedom for others, provided that the original source and author(s) are credited.Carolina Reyes-Puig, Emilio Mancero

Supplementary material 2 Complete translation of the manuscript Data type PDF file Explanation note We present the complete translation of the manuscript into Spanish, including tables and figures. https://binary.pensoft.net/file/779040This dataset is made available under the Open Database License (http://opendatacommons.org/licenses/odbl/1.0/). The Open Database License (ODbL) is a license agreement intended to allow users to freely share, modify, and use this Dataset while maintaining this same freedom for others, provided that the original source and author(s) are credited.Carolina Reyes-Puig, Emilio Mancero

## References

[B1] Armesto LO Señaris JC (2017) Anuros del norte de los andes: Patrones de riqueza de especies y estado de conservación. Papéis Avulsos de Zoologia (São Paulo) 57(39): 491–526. 10.11606/0031-1049.2017.57.39

[B2] Arroyo SB Serrano-Cardozo VH Ramírez-Pinilla MP (2008) Diet, microhabitat and time of activity in a *Pristimantis* (Anura, Strabomantidae) assemblage. Phyllomedusa: Journal of Herpetology 7(2): 109–119. 10.11606/issn.2316-9079.v7i2p109-119

[B3] Boulenger GA (1882) Catalogue of the Batrachia Salientias. Taylor and Francis, London, 256 pp.

[B4] Brito-Zapata D Reyes-Puig C Cisneros-Heredia D Zumel D Ron SR (2021) Description of a new minute frog of the genus *Pristimantis* (Anura: Strabomantidae) from Cordillera del Condor, Ecuador. Zootaxa 5072(4): 351–372. 10.11646/zootaxa.5072.4.3 35390861

[B5] Canedo C Haddad CFB (2012) Phylogenetic relationships within anuran clade Terrarana, with emphasis on the placement of Brazilian Atlantic rainforest frogs genus *Ischnocnema* (Anura: Brachycephalidae). Molecular Phylogenetics and Evolution 65(2): 610–620. 10.1016/j.ympev.2012.07.016 22842090

[B6] Catenazzi A Lehr E (2018) *Pristimantisantisuyu* sp. n. and *Pristimantiserythroinguinis* sp. n., two new species of terrestrial-breeding frogs (Anura, Strabomantidae) from the eastern slopes of the Andes in Manu National Park, Peru. Zootaxa 4394(2): 185–206. 10.11646/zootaxa.4394.2.2 29690369

[B7] Coloma LA Guayasamin JM (2022) William E. Duellman (1930–2022). Phyllomedusa: Journal of Herpetology 21: 103–111.

[B8] Costello MJ May RM Stork NE (2013) Can we name Earth’s species before they go extinct? Science 339(6118): 413–416. 10.1126/science.1230318 23349283

[B9] de Queiroz K (2005) A unified concept of species and its consequences for the future of taxonomy. Proceedings of the California Academy of Sciences 56: 196–215.

[B10] Donthu N Gustafsson A (2020) Effects of COVID-19 on business and research. Journal of Business Research 117: 284–289. 10.1016/j.jbusres.2020.06.008 32536736 PMC7280091

[B11] Duellman WE Lehr E (2009) Terrestrial-breeding frogs (Strabomantidae) in Peru. Natur und Tier Verlag, Münster, 382 pp.

[B12] Duellman WE Pramuk JB (1999) Frogs of the genus *Eleutherodactylus* (Anura: Leptodactylidae) in the Andes of northern Peru. Scientific Papers Natural History Museum, University of Kansas 13: 1–78. 10.5962/bhl.title.16169

[B13] Fox CW Ritchey JP Paine CET (2018) Patterns of authorship in ecology and evolution: First, last, and corresponding authorship vary with gender and geography. Ecology and Evolution 8(23): 11492–11507. 10.1002/ece3.4584 30598751 PMC6303722

[B14] Frost D (2022) Amphibian Species of the World: an Online Reference. Version 6.1. Electronic Database. [Accessible at:] https://amphibiansoftheworld.amnh.org/index.php [accessed 30 January 2022]

[B15] González-Durán GA Targino M Rada M Grant T (2017) Phylogenetic relationships and morphology of the *Pristimantis* leptolophus species group (Amphibia: Anura: Brachycephaloidea), with the recognition of a new species group in *Pristimantis* Jiménez de la Espada, 1870. Zootaxa 4243: 042–074. 10.11646/zootaxa.4243.1.2 28610171

[B16] Guayasamin JM Krynak T Krynak K Culebras J Hutter CR (2015) Phenotypic plasticity raises questions for taxonomically important traits: A remarkable new Andean rainfrog (*Pristimantis*) with the ability to change skin texture. Zoological Journal of the Linnean Society 173(4): 913–928. 10.1111/zoj.12222

[B17] Guayasamin JM Hutter CR Tapia EE Culebras J Peñafiel N Pyron RA Morochz C Funk WC Arteaga A (2017) Diversification of the rainfrog *Pristimantisornatissimus* in the lowlands and Andean foothills of Ecuador. PLoS ONE 12: e0172615. 10.1371/journal.pone.0172615 PMC5362048 28329011

[B18] Guerra García JM Espinosa Torre F García Gómez JC (2008) Trends in taxonomy today: an overview about the main topics in taxonomy. Zoológica Baetica 19: 15–49.

[B19] Hedges SB Duellman WE Heinicke MP (2008) New World direct-developing frogs (Anura: Terrarana): molecular phylogeny, classification, biogeography, and conservation. Zootaxa 1737(1): 1–182. 10.11646/zootaxa.1737.1.1

[B20] Heinicke MP Duellman WE Hedges SB (2007) Major Caribbean and Central American frog faunas originated by ancient oceanic dispersal. Proceedings of the National Academy of Sciences of the United States of America 104(24): 10092–10097. 10.1073/pnas.0611051104 17548823 PMC1891260

[B21] Heinicke MP Duellman WE Trueb L Means DB Macculloch RD Hedges SB (2009) A new frog family (Anura: Terrarana) from South America and an expanded directdeveloping clade revealed by molecular phylogeny. Zootaxa 2211(1): 1–35. 10.11646/zootaxa.2211.1.1

[B22] Heinicke MP Lemmon AR Lemmon EM McGrath K Hedges SB (2018) Phylogenomic support for evolutionary relationships of New World direct-developing frogs (Anura: Terraranae). Molecular Phylogenetics and Evolution 118: 145–155. 10.1016/j.ympev.2017.09.021 28963082

[B23] Hutter CR Guayasamin JM (2015) Cryptic diversity concealed in the Andean cloud forests: Two new species of rainfrogs (*Pristimantis*) uncovered by molecular and bioacoustic data. Neotropical Biodiversity 1(1): 36–59. 10.1080/23766808.2015.1100376

[B24] Illinois Wesleyan University (2022) Edgar Lehr, Ph.D. https://www.iwu.edu/biology/faculty/lehr.html [accessed 3 June 2022]

[B25] INABIO [Instituto Nacional de Biodiversidad] (2022) Colecciones científicas: Herpetología. http://inabio.biodiversidad.gob.ec/colecciones_inabio/ [accessed 3 June 2022]

[B26] IUCN (2022) The IUCN Red List of Threatened Species. https://www.iucnredlist.org [accessed 14 June 2022]

[B27] Jiménez de la Espada M (1870) Fauna neotropicalis species quaedam nondum cognitae. Jornal de Sciências, Mathemáticas, Physicas e Naturaes 3: 57–65.

[B28] Kaiser H Barrio-Amorós CL Rivas GA Steinlein C Schmid M (2015) Five new species of *Pristimantis* (Anura: Strabomantidae) from the coastal cloud forest of the Península de Paria, Venezuela. Journal of Threatened Taxa 7(4): 7047–7088. 10.11609/JoTT.o4197.7047-88

[B29] Lehr E (2007) New eleutherodactyline frogs (Leptodactylidae: *Pristimantis*, *Phrynopus*) from Peru. Bulletin of the Museum of Comparative Zoology 159(2): 145–178. 10.3099/0027-4100(2007)159[145:NEFLPP]2.0.CO;2

[B30] Lehr E Aguilar C Siu-Ting K Carlos Jordán J (2007) Three New Species of *Pristimantis* (Anura: Leptodactylidae) from the Cordillera De Huancabamba in Northern Peru. Herpetologica 63(4): 519–536. 10.1655/0018-0831(2007)63[519:TNSOPA]2.0.CO;2

[B31] Lehr E Lyu S Catenazzi A (2021) A new, critically endangered species of *Pristimantis* (Amphibia: Anura: Strabomantidae) from a mining area in the Cordillera Occidental of northern Peru (Región Cajamarca). Salamandra 57(1): 15–26.

[B32] Lynch JD (1968) Systematic status of some Andean leptodactylid frogs with a description of a new species of *Eleutherodactylus*. Herpetologica 24: 289–300.

[B33] Lynch JD (1971) Evolutionary relationships, osteology, and zoogeography of leptodactyloid frogs. University of Kansas Museum of Natural History. Miscellaneous Publications 53: 1–2.

[B34] Lynch JD (1979) Leptodactylid frogs of the genus *Eleutherodactylus* from the Andes of southern Ecuador. Miscellaneous Publication Museum of Natural History, University of Kansas 66: 1–62. 10.5962/bhl.title.16268

[B35] Lynch JD (1998) New species of *Eleutherodactylus* from the Cordillera Occidental of western Colombia with a synopsis of the distributions of species in western Colombia. Revista de la Academia Colombiana de Ciencias Exactas, Físicas y Naturales 22: 117–148.

[B36] Lynch JD Duellman WE (1980) The *Eleutherodactylus* of the Amazonian slopes of the Ecuadorian Andes (Anura: Leptodactylidae). Miscellaneous Publication Museum of Natural History, University of Kansas 69: 1–86. 10.5962/bhl.title.16222

[B37] Lynch JD Duellman WE (1997) Frogs of the genus *Eleutherodactylus* in western Ecuador, Systematics, ecology, and biogeography. Special Publication Natural History Museum, University of Kansas 23: 1–236. 10.5962/bhl.title.7951

[B38] Lynch JD Rueda-Almonacid JV (1998) Additional new species of frogs (genus *Eleutherodactylus*) from cloud forests of eastern Departamento de Caldas, Colombia. Revista de la Academia Colombiana de Ciencias Exactas, Físicas y Naturales 22: 287–298.

[B39] Malakhov V (2013) A revolution in zoology: New concepts of the metazoan system and phylogeny. Herald of the Russian Academy of Sciences 83(2): 123–127. 10.1134/S1019331613020044

[B40] Mendoza ÁM Ospina OE Cárdenas-Henao H García-R JC (2015) A likelihood inference of historical biogeography in the world’s most diverse terrestrial vertebrate genus: Diversification of direct-developing frogs (Craugastoridae: *Pristimantis*) across the Neotropics. Molecular Phylogenetics and Evolution 85: 50–58. 10.1016/j.ympev.2015.02.001 25681675

[B41] Meneghini R Packer AL Nassi-Calo L (2008) Articles by Latin American authors in prestigious journals have fewer citations. PLoS ONE 3(11): e3804. 10.1371/journal.pone.0003804 PMC2583044 19030227

[B42] Meza-Joya FL Torres M (2016) Spatial diversity patterns of *Pristimantis* frogs in the Tropical Andes. Ecology and Evolution 6(7): 1901–1913. 10.1002/ece3.1968 26929819 PMC4759521

[B43] MUSM [Museo de Historia Natural de la Universidad de San Marcos] (2022) División de Herpetología. https://museohn.unmsm.edu.pe/ [accessed 3 June 2022]

[B44] Ortega-Andrade HM Rojas-Soto OR Valencia JH Espinosa de los Monteros A Morrone JJ Ron SR Cannatella DC (2015) Insights from Integrative Systematics Reveal Cryptic Diversity in *Pristimantis* Frogs (Anura: Craugastoridae) from the Upper Amazon Basin. PLoS ONE 10: e0143392. 10.1371/journal.pone.0143392 PMC4658055 26600198

[B45] Ortega-Andrade HM Rodes Blanco M Cisneros-Heredia DF Guerra Arévalo N López de Vargas-Machuca KG Sánchez-Nivicela JC Armijos-Ojeda D Cáceres Andrade JF Reyes-Puig C Quezada Riera AB Székely P Rojas Soto OR Székely D Guayasamin JM Siavichay Pesántez FR Amador L Betancourt R Ramírez-Jaramillo SM Timbe-Borja B Gómez Laporta M Webster Bernal JF Oyagata Cachimuel LA Chávez Jácome D Posse V Valle-Piñuela C Padilla Jiménez D Reyes-Puig JP Terán-Valdez A Coloma LA Pérez Lara MB Carvajal-Endara S Urgilés M Yánez Muñoz MH (2021) Red List assessment of amphibian species of Ecuador: A multidimensional approach for their conservation. PLoS ONE 16: 1–28. 10.1371/journal.pone.0251027 PMC8101765 33956885

[B46] Padial JM De la Riva I (2009) Integrative taxonomy reveals cryptic Amazonian species of *Pristimantis* (Anura: Strabomantidae). Zoological Journal of the Linnean Society 155(1): 97–122. 10.1111/j.1096-3642.2008.00424.x

[B47] Padial JM Miralles A De la Riva I Vences M (2010) The integrative future of taxonomy. Frontiers in Zoology 7(1): 16. 10.1186/1742-9994-7-16 20500846 PMC2890416

[B48] Padial JM Grant T Frost DR (2014) Molecular systematics of terraranas (Anura: Brachycephaloidea) with an assessment of the effects of alignment and optimality criteria. Zootaxa 3825: 1–132. 10.11646/zootaxa.3825.1.1 24989881

[B49] Páez NB Ron SR (2019) Systematics of *Huicundomantis*, a new subgenus of *Pristimantis* (Anura, Strabomantidae) with extraordinary cryptic diversity and eleven new species. ZooKeys 868: 1–112. 10.3897/zookeys.868.26766 31406482 PMC6687670

[B50] Parise Pinto ÂP Mejdalani G Mounce R Silveira LF Marinoni L Rafael JA (2021) Are publications on zoological taxonomy under attack? Royal Society Open Science 8(2): 201617. 10.1098/rsos.201617 PMC8074659 33972859

[B51] Peters JA (1955) Herpetological type localities in Ecuador. Revista Ecuatoriana de Entomología y Parasitología 2: 335–352.

[B52] Pinto-Sánchez NR Ibáñez R Madriñán S Sanjur OI Bermingham E Crawford AJ (2012) The Great American Biotic Interchange in frogs: Multiple and early colonization of Central America by the South American genus *Pristimantis* (Anura: Craugastoridae). Molecular Phylogenetics and Evolution 62(3): 954–972. 10.1016/j.ympev.2011.11.022 22178362

[B53] Pröhl H Ron SR Ryan MJ (2010) Ecological and genetic divergence between two lineages of Middle American tungara frogs *Physalaemus* (=*Engystomops*) *pustulosus*. BMC Evolutionary Biology 10(1): 2–18. 10.1186/1471-2148-10-146 20482771 PMC2882927

[B54] Pyron AR Wiens JJ (2011) A large-scale phylogeny of Amphibia including over 2800 species, and a revised classification of extant frogs, salamanders, and caecilians. Molecular Phylogenetics and Evolution 61(2): 543–583. 10.1016/j.ympev.2011.06.012 21723399

[B55] R Core Team (2022) R: A language and environment for statistical computing. R Foundation for Statistical Computing, Vienna. https://www.R-project.org/

[B56] Salerno PE Páez-Vacas M Guayasamin JM Stynoski JL (2019) Male principal investigators (almost) don’t publish with women in ecology and zoology. PLoS ONE 14: 1–14. 10.1371/journal.pone.0218598 PMC6583967 31216351

[B57] Ramírez-Castañeda V (2020) Disadvantages in preparing and publishing scientific papers caused by the dominance of the English language in science: The case of Colombian researchers in biological sciences. PLoS ONE 15(9): 1–15. 10.1371/journal.pone.0238372 PMC7494110 32936821

[B58] Reyes-Puig C Yánez-Muñoz MH Ortega JA Ron SR (2020) Relaciones filogenéticas del subgénero *Hypodictyon* (Anura: Strabomantidae: *Pristimantis*) con la descripción de tres especies nuevas de la región del Chocó. Revista Mexicana de Biodiversidad 91(0): 913013. 10.22201/ib.20078706e.2020.91.3013

[B59] Rock KN Barnes IN Deyski MS Glynn KA Milstead BN Rottenborn ME Andre NS Dekhtyar A Dekhtyar O Taylor EN (2021) Quantifying the Gender Gap in Authorship in Herpetology. Herpetologica 77(1): 1–13. 10.1655/0018-0831-77.1.1

[B60] Ron SR Carrión J Caminer MA Sagredo Y Navarrete MJ Ortega JA Varela-Jaramillo A Maldonado-Castro GA Terán C (2020) Three new species of frogs of the genus *Pristimantis* (Anura, Strabomantidae) with a redefinition of the *P.lacrimosus* species group. ZooKeys 993: 121–155. 10.3897/zookeys.993.53559 33262676 PMC7683497

[B61] Ron S Merino-Viteri A Ortiz D (2022) Anfibios del Ecuador. Version 2022.0. Museo de Zoología. https://bioweb.bio/faunaweb/amphibiaweb [accessed 3 January 2022]

[B62] Salerno PE Páez-Vacas M Guayasamin JM Stynoski JL (2019) Male principal investigators (almost) don’t publish with women in ecology and zoology. PLoS ONE 14(6): e0218598. 10.1371/journal.pone.0218598 PMC6583967 31216351

[B63] Slobodian V Soares KDA Falaschi RL Prado LR Camelier P Guedes TB Leal LC Hsiou AS Del-Rio G Costa ER Pereira KRC D’Angiolella AB de A Sousa S Diele-Viegas LM (2021) Why we shouldn’t blame women for gender disparity in academia: Perspectives of women in zoology. Zoologia 38: 1–9. 10.3897/zoologia.38.e61968

[B64] Taucce PPG Nascimento JS Trevisan CC Leite FSF Santana DJ Haddad CFB Napoli MF (2020) A new rupicolous species of the *Pristimantisconspicillatus* group (Anura: Brachycephaloidea: Craugastoridae) from Central Bahia, Brazil. Journal of Herpetology 54(2): 24–257. 10.1670/19-114

[B65] Torres-Carvajal O Pazmiño-Otamendi G Ayala-Varela F Salazar-Valenzuela D (2022) Reptiles del Ecuador. https://bioweb.bio/faunaweb/reptiliaweb [accessed 3 June 2022]

[B66] Wägele H Klussmann-Kolb A Kuhlmann M Haszprunar G Lindberg D Koch A Wägele JW (2011) The taxonomist-an endangered race. A practical proposal for its survival. Frontiers in Zoology 8(1): 1–7. 10.1186/1742-9994-8-25 22029904 PMC3210083

[B67] West JD Jacquet J King MM Correll SJ Bergstrom CT (2013) The role of gender in scholarly authorship. PLoS ONE 8(7): 1–6. 10.1371/journal.pone.0066212 PMC3718784 23894278

[B68] Woolbright LL (1985) Patterns of nocturnal movement and calling by the tropical frog *Eleutherodactylus* coqui. Herpetologica 41(1): 1–9.

[B69] Yánez-Muñoz MH Meza-Ramos PA Cisneros-Heredia DF Reyes-Puig JP (2010) Descripción de tres nuevas especies de ranas del género *Pristimantis* (Anura: Terrarana: Strabomantidae) de los bosques nublados del Distrito Metropolitano de Quito, Ecuador. ACI Avances en Ciencias e Ingenierías 2(3): B16–B27. 10.18272/aci.v2i3.40

