# Supplementary material for: ﻿Beyond the species name: an analysis of publication trends and biases in taxonomic descriptions of rainfrogs (Amphibia, Strabomantidae, Pristimantis)
Source: Zookeys. 2022 Dec 7;1134:73–100. doi: 10.3897/zookeys.1134.91348 (PMC9836588; doi:10.3897/zookeys.1134.91348)
Supplement: Supplementary material 2 — Complete translation of the manuscript [file zookeys-1134-073_article-91348__-s002.pdf]

Más allá del nombre de la especie: un análisis de las tendencias de publicación y los sesgos en las descripciones taxonómicas de ranas de la lluvia (Amphibia, Strabomantidae, *Pristimantis*)

Carolina Reyes-Puig[1,2,3] y Emilio Mancero [4]

[1]Universidad San Francisco de Quito USFQ, Instituto iBIOTROP, Museo de Zoología & Laboratorio de Zoología Terrestre, Quito, Ecuador

[2]Universidad San Francisco de Quito USFQ, Colegio de Ciencias Biológicas y Ambientales COCIBA, Quito, Ecuador

[3]Instituto Nacional de Biodiversidad, Unidad de Investigación, Quito, Ecuador

[4]Biology Department, Grand Valley State University, Allendale, MI 49401, USA

Autora correspondiente: creyesp@usfq.edu.ec

Resumen

Las ranas de la lluvia del género *Pristimantis* es uno de los grupos de vertebrados más diversos, con una variedad de modos reproductivos y estrategias que impulsan su éxito en la colonización de nuevos hábitats. La tasa de especies de *Pristimantis* descubiertas anualmente ha aumentado continuamente durante los últimos 50 años, estableciendo la notable diversidad encontrada en este género. En este artículo, examinamos los detalles de las publicaciones que describen nuevas especies en el grupo, incluida la autoría, el año, el idioma, la revista, el género, las colecciones científicas y otros detalles. Analizamos y extrajimos información detallada sobre las descripciones de 591 especies de *Pristimantis* publicadas hasta la fecha (junio 2022). John D. Lynch y William E. Duellman son los autores más prolíficos, pero los investigadores latinoamericanos han ampliado y continuado los procesos de descripción desde la década de 1990. El idioma más común utilizado para las descripciones es el inglés, seguido del español. La gran mayoría de los autores han descrito una sola especie. La mayor proporción de autores que han participado en las descripciones es de nacionalidad ecuatoriana. Ecuador es el país con la tasa de descripción más alta por año (tasa de crecimiento del 3,9%). Solo el 20% de las contribuciones han incluido a mujeres y solo el 2% las ha presentado como autoras principales. El 36,8% de las especies descritas se encuentran en las categorías No evaluadas o Datos insuficientes de la lista roja mundial de la UICN. Destacamos la importancia de potenciar las descripciones en español y fomentar la inclusión de mujeres investigadoras en la taxonomía de *Pristimantis*. En general, si continúan las

tendencias actuales en las descripciones de *Pristimantis*, en 10 años se podría esperar un total de 770 o más especies descritas.

Introducción

Pristimantis Jimenez de la Espada, 1870 es un clado de ranas de desarrollo directo provenientes del nuevo mundo, pertenecientes a la familia Strabomantidae, orden Anura, clase Amphibia, filo Chordata. Es el género de vertebrados terrestres más especioso con 591 especies descritas hasta la fecha (Hedges et al. 2008; Frost 2022). Un mayor énfasis en técnicas moleculares, acústicas y osteológicas combinadas con un marcado incremento en muestreos ha dado lugar a un incremento en descripciones de nuevas especies en años recientes, fomentando el progreso en investigación para entender su taxonomía y sistemática (Padial et al. 2010; Hutter & Guayasamin 2015; Kaiser et al. 2015, González-Durán et al. 2017; Reyes-Puig et al. 2020).

La descripción más temprana en este género corresponde al trabajo de Jiménez de la Espada, con la descripción del género tipo *Pristimantis galdi* (Jiménez de la Espada 1870). El género luego fue puesto en sinonimia con *Hylodes* sensu lato por Boulenger (1882), y luego sinonimizado como *Eleutherodactylus* por Peters (1955). *Cyclocephalus*, *Pseudohyla* y *Trachyphrynus* fueron también sinonimizados con *Eleutherodactylus* por Lynch (1968, 1971). Henicke et al. (2007) luego removió la sinonimia de *Eleutherodactylus* con el apoyo de evidencia molecular. Este vasto y fenotípicamente diverso genero ha sido sujeto a posteriores análisis moleculares confirmando su monofilia y estado como un taxón hermano a *Lynchius*, *Oreobates* y *Phyrnopus*, con *Yunganastes* siendo sugerido también (Pyron & Wiens 2011; Canedo & Haddad 2012; Pinto-Sánchez et al. 2012). Varios grupos distintos pueden ser hallados dentro de este género, originalmente descritos en base a extensiva evidencia morfológica, con posteriores revisiones apoyadas por análisis moleculares (Lynch and Duellman 1997; Hedges et al. 2008; Padial et al. 2014; Pinto-Sánchez et al. 2012; Padial et al. 2014; Mendoza et al. 2015). En la actualidad hay 13 grupos de especies reconocidos, siendo *P. conspicillatus* el más grande con 36 especies (Padial et al. 2014; González-Durán 2017; Taucce et al. 2020; Reyes-Puig et al. 2020). Varias otras especies continúan sin ser asignadas a algún grupo puesto a que son demostrablemente no-monofiléticas y por lo tanto de poco interés taxonómico hasta que datos de secuencias de ADN del género entero estén disponibles (Hedges et al. 2008; Padial et al. 2014).

Los miembros de este género son notables por depositar sus huevos en hábitats terrestres, en donde los embriones se desarrollan directamente como ranas sin pasar por la etapa acuática de su ciclo de vida (Woolbright 1985; Duellman and Lehr 2009). Ensamblajes de especies de *Pristimantis* son comunes, ya que su morfología y por lo tanto su comportamiento y actividades ecológicas son considerablemente similares (Arroyo et al. 2008). Se caracterizan por ser insectívoros generalistas, eligiendo presas en base a disponibilidad y tamaño (Lynch and Duellman 1997; Arroyo et al. 2008). Los individuos son predominantemente arbóreos y nocturnos, usualmente trepados en hojas a alturas bajo 200 cm. Ya que su biología reproductiva desalienta el congregarse en charcas, los machos de este género prefieren vocalizar ruidosamente desde el suelo o alguna percha en busca de una pareja (Lynch and Duellman 1997; Duellman and Lehr 2009). El género está ampliamente distribuido en el Nuevo Mundo y se considera el más extenso entre los anfibios Neotropicales, con especies comúnmente halladas en bosques tropicales y subtropicales en América del Sur hasta el sur de América Central (Lynch and Duellman 1997; Pinto-Sánchez et al. 2012; Meza-Joya and Torres 2016; Armesto and Señaris 2017). El grupo muestra niveles particularmente altos de diversidad y endemismo a lo largo de ambientes húmedos a una altura aproximada de 4000m en los Andes y en tierras bajas aledañas como el Chocó sudamericano (Heinicke et al. 2007; Meza-Joya and Torres 2016; Armesto and Señaris 2017; Reyes-Puig et al., 2020). El continuo incremento en el número de especies descritas dentro de este género sugiere una riqueza de especies mucho mayor a la esperada, con sutiles diferencias en comportamiento y fisiología reflejando distintas trayectorias evolutivas (de Queiroz 2005; Hutter & Guayasamin 2015).

Las condiciones que llevaron a tan alta tasa de diversificación en *Pristimantis* no son completamente entendidas. Familias de ranas de desarrollo directo divergieron rápidamente durante el Cenozoico temprano y medio, favoreciendo una amplia dispersión a lo largo de un rango de hábitats en América del Sur dando lugar a una rápida acumulación de aislamiento genético poblacional (Henicke et al. 2007; Henicke et al. 2009; Pröhl et al. 2010). Un punto importante en la radiación de este género se halla entre 1000 y 3000m en el Noroeste de los Andes (Mendoza et al. 2015). Las complejas dinámicas biogeográficas en el área no solo apoyaron la especiación alopátrica, sino que también facilitaron la dispersión de especies de tierras bajas durante el Paleoceno y Plioceno, dando lugar a un patrón de especiación particularmente

adecuado para originar linajes crípticos estrechamente relacionados (Lynch and Duellman 1997; Mendoza et al. 2015).

Durante los últimos 20 años, el aumento en las tasas de descripción de especies en América del Sur ha reflejado la alta diversidad y endemismo de *Pristimantis*, destacando estos patrones de especiación aún poco conocidos. Dada la naturaleza críptica de los miembros de este género, el trabajo para descubrir nuevas especies parece estar lejos de terminar. Aunque muchas especies también pueden exhibir un alto grado de plasticidad (Guayasamin et al. 2015), desentrañar estas características en un contexto taxonómico sigue siendo un desafío.

A pesar de que este género es tan diverso y la investigación taxonómica y sistemática continúa de año en año, la información sobre patrones y tendencias de publicación es escasa. Los sesgos asociados con el idioma de publicación y el género aún no se han explorado. Sin embargo, dentro de las ciencias biológicas y específicamente dentro de la Zoología, históricamente los estudios en esta rama se han caracterizado por estar dominados por hombres, generando limitaciones en las carreras actuales de muchas mujeres (Slobodian 2021). Del mismo modo, se ha demostrado que, por ejemplo, en Ecología y Zoología, la proporción de investigadores principales que publican con mujeres es menor en comparación con la proporción de hombres (Salerno et al. 2020). Por otro lado, el idioma de publicación sigue dominado por el idioma inglés, aunque ha habido acercamientos para mejorar la transmisión de la ciencia para que pueda ser difundida localmente (Ramíres-Castañeda 2020). Los investigadores latinoamericanos no solo suelen estar presionados para publicar en inglés, sino también para hacerlo con colegas de países desarrollados, cuestiones que suelen estar relacionadas con la cantidad de citas que puede recibir un artículo. (Meneghini et al. 2008). Por lo tanto, en este artículo pretendemos profundizar en los detalles de los sesgos y tendencias en la publicación de descripciones de nuevas especies de *Pristimantis* mediante la realización de una revisión detallada de todos los parámetros de descripción, enfatizando la ubicación, la autoría (incluido el género), la colección científica y el idioma.

Materiales y Métodos

Seguimos la propuesta de Hedges et al. (2008) y Heinicke et al. (2018) para la clasificación familiar de *Pristimantis* La lista actualizada de las especies descritas formalmente hasta la fecha se extrajo de la página web Amphibian Species of the World del American Museum of Natural History (Frost, 2022). El 14 de junio de 2022, la lista de *Pristimantis* contenía 591 especies descritas. A partir de esta lista, generamos una base de datos detallada para cada especie, extrayendo los datos específicos disponibles en la descripción original y en la mencionada página web.

Construimos una base de datos con los siguientes campos: identificador único de la especie, especie, revista, año de publicación, primer autor, autores, género, nacionalidad de los autores, autor correspondiente, género, país de descripción, localidad tipo, holotipo, sinonimia, grupo de especies (si procede), distribución, idioma de la descripción, afiliaciones institucionales, estado de conservación y tipo de descripción. Para más detalles, véase el material suplementario 1. Calculamos la tasa de crecimiento de las descripciones globales y la de los tres países con mayor número de descripciones (es decir, Ecuador, Colombia y Perú). Para representar mejor las diferentes tendencias históricas observadas en la descripción de *Pristimantis*, las tasas de crecimiento se dividen en dos períodos: El primero desde 1958 - 1989, cuando las descripciones comienzan a ser más constantes, y el segundo desde 1990 - 2021. Debido a un aumento significativo de las descripciones en ese momento, se considera también el período que va de 2010 a 2021. Esto se debe principalmente a que pretendemos identificar una tasa de descripción realista con los últimos avances en la taxonomía y la sistemática del género. Además del hecho de que el número de investigadores que trabajan actualmente en la taxonomía de *Pristimantis* es mayor que en períodos anteriores (Material Suplementario 1). No hemos considerado los periodos anteriores a 1958 porque las descripciones hasta esa fecha no muestran un aumento considerable.

Realizamos la búsqueda de información sobre el género y la nacionalidad de los autores a través de la exploración individual que permite Google. Las categorías de amenaza se obtuvieron de la Lista Roja de la UICN (UICN 2021). Una vez que llenamos la base de datos completa, limpiamos y homogeneizamos la base de datos principalmente para los nombres de los autores, instituciones y revistas, para evitar errores tipográficos y campos duplicados. Para establecer la importancia del aumento de las mujeres que

participan en las descripciones de *Pristimantis* a lo largo de los años, utilizamos modelos lineales generalizados (GLM). Estos incluyen una distribución de error cuasi-binomial, necesaria dada la naturaleza de los datos (es decir, datos de proporcionalidad dicotómica y sobredispersión). Consideramos los años como la variable dependiente y la proporción de autoras como la variable independiente, ya que nos interesaba identificar una tendencia en la proporción de mujeres y el tiempo. Hicimos un GLM del total de autoras y uno específico para cada país de los siete con mayor diversidad de *Pristimantis* (Ecuador, Colombia, Perú ,Venezuela, Brasil, Panamá y Bolivia), consideramos los datos desde 1970, que es cuando comenzaron a aparecer mujeres en las descripciones de *Pristimantis*. Todo el manejo, limpieza y análisis de la base de datos lo hicimos con el software estadístico R (R Core team 2022) y los paquetes "tidyverse", "forcats" y "gridExtra".

Las descripciones de *Pristimantis* empezaron en 1958 con *Pristimantis* conspicillatus (bajo la sinonimia de Hylodes conspicillatus, Lithodytes conspicillatus y más tarde Eleutherodactylus conspicillatus). Durante un siglo las descripciones de este grupo se mantuvieron relativamente estables, con menos de 4 especies descritas de media cada 10 años (Fig. 1). A partir de los años 60, las descripciones del grupo aumentaron significativamente con varios picos hacia los años 70 pero con uno en particular hacia 1980. El número de descripciones cada cinco años aumentó en relación con el rango entre 1958-1970, con una media de cinco a catorce descripciones entre 1978 y 1996. Después de este año, los picos más notables de nuevas descripciones se restringieron a los años 1997, 1998, 1999, 2007, 2019, 2020 y 2021 (Fig. 1A). El aumento significativo de las descripciones de nuevas especies en este grupo se intensificó después de 1978 y la curva de acumulación de descripciones parece seguir aumentando hasta la fecha actual, con un total de 591 especies formalmente descritas (Fig. 1B). El período con las mayores tasas anuales de descripción de *Pristimantis* se encuentra entre 1958-1989. Para Ecuador, el periodo con mayor tasa de descripción se encuentra entre 2010-2021, para Colombia entre 1958-1989 y para Perú entre 1990-2021 (Tabla 1).

El área de distribución del género *Pristimantis* se extiende desde Panamá hasta Perú. Ecuador es el país con el mayor número de especies descritas con 212 descripciones totales, seguido de Colombia, Perú y Venezuela. Otros países dentro del área de distribución de este género reportan menos de 10 descripciones (Fig. 2A). A partir de

los años 60, las descripciones ocurren predominantemente en Ecuador, Colombia y Perú, con al menos dos picos de descripciones superiores al promedio de diez nuevas especies descritas por año (Fig. 2B). Ecuador presenta tres picos importantes de descripción de especies (i.e., 1979, 1980 y 2019), con más de 15 nuevas especies descritas por año; Colombia alcanza un pico en 1998 llegando a 20 descripciones en un solo año, estableciendo un récord en descripciones para este género; y finalmente, Perú alcanza dos picos (i.e., 1999 y 2007) llegando a 13 y 15 descripciones de especies. (Fig. 2C).

Un total de 320 investigadores están formalmente reconocidos como autores y coautores (Material Suplementario 1). Casi el 46% de ellos han descrito una sola especie, otro 46% han descrito entre dos y siete especies, el 6,5% de los autores han descrito entre ocho y treinta especies y finalmente sólo el 0,6% de los autores han descrito más de 50 especies (Fig. 3A). J. D. Lynch es el autor con el mayor número de especies recién descritas, con 195 especies. Le siguen W. E. Duellman con 82, E. Lehr y M. Yánez-Muñoz con 36 cada uno. Detalles sobre los 20 autores con más descripciones en la Tabla 2. Por otro lado, dentro de todas las colecciones científicas donde se ha depositado el material tipo, las cuatro instituciones con mayor número de holotipos son el Museo de Historia Natural de la Universidad de Kansas (KU), el Museo de Historia Natural del Instituto de Ciencias Naturales de la Universidad Nacional de Colombia (ICN), el Museo de Zoología de la Pontificia Universidad Católica del Ecuador (QCAZ), y la División de Herpetología del Instituto Nacional de Biodiversidad, antiguo Museo Ecuatoriano de Ciencias Naturales (DHMECN). La KU y el ICN albergan el 35% del material tipo, y el QCAZ y el DHMECN tienen el 12,5% de los especímenes tipo del género (Fig. 3B). El resto de las colecciones albergan una proporción menor de ejemplares que los mencionados (Fig. 3B, Material Suplementario 1).

Identificamos una variedad de ocho idiomas utilizados para la descripción de nuevas especies de *Pristimantis* (Fig. 4A, Tabla 3), sin embargo, los dominantes han sido el inglés y el español. El idioma inglés ha dominado las descripciones desde los años 60 hasta la actualidad. El número de descripciones publicadas en español comenzó a aumentar durante los años 80, aunque sigue siendo menor que las publicadas en inglés (Fig. 4A, Tabla 3). Por el contrario, hay más de 20 nacionalidades de investigadores que han participado como autores y coautores en las descripciones de nuevas especies de

Pristimantis (Fig. 4B). Los investigadores de EE.UU. han dominado significativamente, no sólo el número total de descripciones, sino también el número de descripciones anuales hasta 2002. A partir de este año y hasta el presente, la participación de los autores ecuatorianos ha aumentado en mayor número en comparación con sus pares colombianos, peruanos y venezolanos (Fig. 4B). Es importante mencionar que del total de autores y coautores que han participado en las descripciones, el 20,9% han sido ecuatorianos, seguidos por estadounidenses y colombianos Tabla 3.

Desde la perspectiva de género, el 80% de los autores que han participado en las descripciones son investigadores varones y el 20% mujeres (Fig. 5-6). A pesar del aumento del número de descripciones con participación femenina a partir de los años 90, la participación de las mujeres en el proceso de descripción sigue siendo considerablemente inferior a la de sus homólogos masculinos (Fig. 5A). Ecuador es el país con mayor porcentaje de descripciones que incorporan mujeres investigadoras con casi un 18,8%, mientras que Colombia, Perú, Venezuela y Brasil tienen porcentajes menores (Fig. 5 B). En cambio, en cuanto a la autoría principal (es decir, primer autor o autor correspondiente), la comparación se vuelve mucho más desproporcionada. Casi el 50% de los autores masculinos han sido acreditados como autores principales, mientras que sólo el 2% de las autoras han desempeñado este papel (Fig. 6, Tabla 5). Hemos detectado una pendiente significativa entre la proporción de autoras que han participado en las descripciones de todo *Pristimantis* y los años (estimación = 0,03, SE = 0,01 t = 2,2, p = 0,02) (Fig. 7). De los cuatro países con mayor número de especies de *Pristimantis*, Ecuador fue el único con una pendiente positiva significativa entre la proporción de autoras y el tiempo (estimación = 0,09, SE = 0,02, t = 3,8, p = < 0 001), mientras que por el contrario Colombia (estimación = 3,7 *10-3, SE = 0,01, t = 0,2 , p = 0,8), Perú (estimación = -0,01, SE = 0,02, t = 3,8 , p = 0,5) y Venezuela (estimación = -0,01, SE = 0,03, t = -0,5 , p = 0,7) no mostraron ninguna correlación entre estas variables (Fig. 7).

En relación con las revistas en las que se han publicado las descripciones de nuevas especies del género, Zootaxa y la Revista de la Academia Colombiana de Ciencias, Exactas, Físicas y Naturales han publicado el mayor número de descripciones con un 20,6% del total de especies descritas. Otras revistas como Herpetologica, Zookeys y Miscellaneus Publication of Museum of Natural History of University of Kansas han

publicado el 15,7% de las descripciones de *Pristimantis* (Fig. 8A). Desde el inicio de las descripciones de este diverso género hasta la primera década del 2000, las descripciones se han basado principalmente en la morfología. Los análisis filogenéticos se incorporaron gradualmente a las descripciones en los últimos 12 años, lo que llevó a una reducción significativa de las descripciones de nuevas especies de *Pristimantis* basadas únicamente en la morfología (Fig. 8B).

En cuanto al estado de conservación de las especies de *Pristimantis*, el 24% están categorizadas por la Lista Reed de la UICN como de Preocupación Menor, el 31% están amenazadas (es decir, CR, EN y VU) y el 36,8% están No Evaluadas o con Datos Insuficientes (Fig. 9A). El país con el mayor número de especies No evaluadas es Ecuador (es decir, el 39% de todas las especies ecuatorianas). Perú y Venezuela albergan los mayores porcentajes de especies bajo la categoría de Datos Insuficientes, 31,8% y 42,8% del total de sus especies respectivamente. En Ecuador y Colombia, al menos el 30% de las especies de *Pristimantis* están bajo alguna forma de amenaza (Fig. 9B).

Discusión

Autores más prolíficos y países con mayor número de descripciones

Las contribuciones hechas por John D. Lynch and Willman E. Duellman al avance de la taxonomía y sistemática de *Pristimantis* desde los setentas son indiscutibles. Sus contribuciones más significativas se centran en grandes compendios que incluyen análisis de patrones de distribución y avances en la sistemática y descripción de varias especies nuevas (Lynch 1979; Lynch and Duellman 1980; Lynch and Duellman 1997; Duellman and Pramuk 1999). Su trabajo se centró inicialmente en la vertiente oriental de Ecuador (Lynch 1979; Lynch and Duellman 1980) y más tarde, hacia los años 90, sus intereses se desplazaron hacia el piedemonte colombiano y peruano (Lynch 1998; Duellman and Pramuk 1999). Los trabajos más representativos y los años más productivos para la descripción de nuevas especies de este género fueron: 1979 con “Leptodactylid frogs of the genus *Eleutherodactylus* from the Andes of southern Ecuador” by J. D. Lynch y la descripción de 16 especies nuevas; 1980 con “*Eleutherodactylus* of the Amazonian slopes of the Ecuadorian Andes (Anura: Leptodactylidae)” by Lynch and Duellman, que incluye 12 nuevas especies; 1997 con

“Frogs of the genus *Eleutherodactylus* in western Ecuador” by Lynch and Duellman; 1998 with “New frogs of the genus *Eleutherodactylus* from the eastern flank of the northern Cordillera Central of Colombia” by Lynch and Rueda-Almonacid and “New species of *Eleutherodactylus* from the Cordillera Occidental of western Colombia with a synopsis of the distributions of species in western Colombia” by Lynch, en los cuales nueve especies nuevas son descritas; 1999 con “Frogs of the genus *Eleutherodactylus* (Anura: Leptodactylidae) in the Andes of northern Peru” by Duellman and Pramuk, describiendo más de 15 especies nuevas; 2007 con “Three new species of *Pristimantis* (Anura: Leptodactylidae) from the Cordillera de Huancabamba in northern Peru” y “New eleutherodactyline frogs (Leptodactylidae: *Pristimantis*, *Phrynopus*) from Peru” both by Lehr con tres y cuatro especies nuevas cada una; 2019 con “Systematics of *Huicundomantis*, a new subgenus of *Pristimantis* (Anura, Strabomantidae) with extraordinary cryptic diversity and eleven new species” by Páez y Ron la cuál adicionó 11 nuevas especies a Ecuador después de casi 40 años de una contribución que incluye descripciones conjuntas de *Pristimantis*, como las publicadas anteriormente por Lynch and Duellman (Lynch 1979; Lynch and Duellman 1980); finalmente 2021 con varias descripciones de diferentes autores (véase el material suplementario 1). Si se mantienen las tendencias actuales en la tasa de crecimiento de las descripciones anuales de Pristimantis y tomando en consideración la historia temporal completa de las descripciones de cada país, se espera que el número total de especies aumente en los próximos 10 años a ~777 especies descritas, con ~ 299 en Ecuador, ~217 en Colombia y ~153 en Perú, ~73 en Venezuela, ~22 en Brasil, ~11 en Panamá y ~6 en Bolivia.

¿Por qué Ecuador lidera en el número de nuevas especies de *Pristimantis* descritas?

Las contribuciones de Lynch y Duellman en la definición del grupo como un género diverso y altamente endémico se convirtieron en una gran influencia para los investigadores locales, lo que condujo a más descubrimientos, particularmente alrededor de Ecuador, Colombia y Perú (Lynch 1979; Lynch y Duellman 1980; Lynch y Duellman 1997). A pesar de ser el más pequeño por área de estos tres, Ecuador presenta tanto el mayor número de descripciones de nuevas especies de *Pristimantis* como la mayor riqueza del género, seguido por Colombia y Perú, lo que sugiere que la diversidad conocida del género está subestimada en estos países (Frost 2022; Ron et al. 2022). Además, de los seis picos de nuevas especies descritas por año, tres corresponden a

Ecuador, evidenciando un gran compromiso con los esfuerzos taxonómicos que se realizan en esta región.

John D. Lynch, herpetólogo norteamericano, es el taxónomo con mayor número de especies descritas de este género con un total de 149 descripciones. Tras trabajar 30 años en la Universidad de Nebraska-Lincoln, a partir de 1997 asumió el cargo de profesor asociado y conservador de herpetología en el Instituto de Ciencias Naturales de la Universidad Nacional de Colombia. William E. Duellman, destacado zoólogo norteamericano que fue conservador emérito de la División de Herpetología del Museo de Historia Natural de la Universidad de Kansas (Coloma y Guayasamin 2022), es el segundo taxónomo más productivo, con 82 especies descritas formalmente. Le siguen en el ranking autores como Edgar Lehr, M. H. Yánez-Muñoz y Santiago R. Ron tomando el relevo de Lynch y Duellman, los tres responsables del 15% de las descripciones totales del género. Edgar Lehr es herpetólogo de la Universidad de Illinois y su trabajo se ha centrado en la sistemática de anfibios, principalmente de Perú (Illinois Wesleyan University 2022). Mario H. Yánez-Muñoz del Instituto Nacional de Biodiversidad y Santiago R. Ron, profesor y conservador del Museo de Zoología QCAZ, se han centrado en la taxonomía y sistemática del género en las laderas y tierras bajas andinas de Ecuador durante los últimos 15 años (Ron et al. 2020, INABIO 2022).

Los dos primeros puestos de los taxónomos más prolíficos para *Pristimantis* están ocupados por investigadores norteamericanos, que representan el 46,8% de la diversidad conocida del género. Sin embargo, entre los 20 autores con más descripciones, el 70% son autores latinoamericanos que siguen los pasos de Lynch y Duellman. El aumento de investigadores latinoamericanos interesados en el género surge de la potenciación de la ciencia y la biodiversidad locales, impulsando un mayor interés a medida que se cumplen los objetivos de investigación. Aunque en los últimos años ha aumentado el número de investigadores interesados en describir nuevas especies de *Pristimantis*, la mayoría de ellos describen entre una y cuatro especies, lo que se refleja en el número de autores que participan en las publicaciones (Material Suplementario 1, por ejemplo, Guayasamin et al. 2017).

Lenguaje de descripción de la especie

El inglés y el español son los idiomas dominantes para las descripciones de las especies de *Pristimantis*. Desde una perspectiva temporal, todo el trabajo desarrollado principalmente por Lynch y Duellman (Lynch y Duellman 1980, Lynch y Duellman 1997, Duellman y Pramuk 1999, Duellman y Lehr 2009) se correlaciona con el idioma, las colecciones científicas, los años de producción, etc. No obstante, aunque desde 1980 se ha producido un aumento del número de descripciones en español, los últimos años se han caracterizado por un incremento del número de descripciones publicadas en inglés. Lo irónico de esta relación es que la mayoría de los investigadores actuales del género son latinoamericanos de habla hispana, publicando en inglés principalmente para otros hispanohablantes. Incluso este artículo entra dentro de este enigma. Sin embargo, nuestro objetivo es visualizar las especificidades de las descripciones en *Pristimantis*, resaltando patrones y tendencias para visualizar a un público más amplio la información que hay detrás de una nueva descripción en un grupo tan diverso. Además, como sugiere Ramírez-Castañeda (2020), adjuntamos una traducción completa al español de este manuscrito como Material Suplementario 2.

La publicación de resultados en inglés está directamente relacionada con las presiones de las instituciones académicas para publicar en revistas de alto impacto, donde el inglés está establecido como idioma oficial de publicación. Publicar en inglés para los investigadores latinoamericanos puede ser un proceso largo, exigente y estresante (Ramírez-Castañeta 2020). A pesar de que el inglés es el idioma común para comunicar la ciencia, la incorporación del español para los grupos taxonómicos distribuidos geográficamente en los países latinoamericanos podría ser también una alternativa valiosa para la difusión de resultados. Por lo tanto, son necesarios mecanismos que promuevan el avance de las investigaciones en *Pristimantis* publicadas en español en revistas especializadas (por ejemplo, Fig. 7).

Nacionalidad de los autores

El número de investigadores ecuatorianos es proporcionalmente mayor que el de otras nacionalidades, como lo demuestra la activa participación de autores ecuatorianos describiendo nuevas especies de *Pristimantis* durante los últimos años (e.g., Yánez-Muñoz et al. 2010, Páez y Ron 2019, Reyes-Puig et al. 2020, Ron et al., 2020). Sin embargo, también cabe mencionar que el número de autores por descripción es mayor

en comparación con las descripciones de la década de 1980 (por ejemplo, Lynch y Duellman 1980). En la actualidad hay descripciones con hasta nueve autores (por ejemplo, Ron et al. 2020, véase el Material Suplementario 1), el número medio de autores en los años 80 está entre 1 y 2, mientras que durante la última década el número medio de autores que participan en las descripciones está entre 5 y 6. En muchas de estas descripciones con varios autores, la mayoría de los autores individuales tienen tasas de descripción muy bajas, mientras que el autor principal es un investigador experimentado en el estudio de la taxonomía y la sistemática del género. En este trabajo también identificamos un vacío en la presencia de taxónomos de *Pristimantis* en Perú, en comparación con Ecuador y Colombia. La inclusión y empoderamiento de los científicos peruanos parecería necesaria para incrementar el estudio de las especies de la selva en su territorio, el cual ha sido liderado principalmente por autores extranjeros (por ejemplo, Duellman y Pramuk 1999, Lehr 2018, Lehr et al. 2021) (los detalles de los autores, años y descripciones se pueden descargar en el Material Suplementario 1).

Colecciones del museo de historia natural

Debido al extenso trabajo de John D. Lynch y William E. Duellman, los museos en los que están depositados los holotipos resultantes de sus descripciones son los que actualmente poseen la mayor cantidad de material tipo de *Pristimantis* (es decir, KU e ICN). Por otro lado, el QCAZ es una institución con más de 40 años dedicados a la salvaguarda e investigación de la biodiversidad ecuatoriana y se ha posicionado como un referente internacional en cuanto a la gestión y acceso abierto a las colecciones científicas, no sólo de anfibios sino también de otros vertebrados. Esta institución destaca por albergar holotipos de 44 especies de *Pristimantis*. (Ron et al. 2022, Torres-Carvajal et al. 2022). Otras instituciones sudamericanas que conservan un gran número de holotipos son el DHMECN y el MUSM (Museo de Historia Natural, Universidad Nacional Mayor de San Marcos). El DHMECN del Instituto Nacional de Biodiversidad se ha centrado en la taxonomía y sistemática de la herpetofauna ecuatoriana durante los últimos 15 años, principalmente en *Pristimantis* de las laderas andinas de Ecuador (INABIO 2022). La División de Herpetología del MUSM tiene una larga trayectoria en la investigación de la herpetofauna peruana desde 1946. Sin embargo, desde 2007 su producción científica relacionada con las descripciones de nuevas especies del género ha disminuido (MUSM 2022). Además, el acceso virtual a sus colecciones y bases de datos es limitado en comparación con las colecciones ecuatorianas (Ron et al. 2022).

Género de los autores

Históricamente, la Zoología ha sido un campo dominado por los hombres. Las limitaciones a las que se enfrentaban las mujeres para acceder y practicar la ciencia antes del siglo XX han llevado a que el estudio de los animales sea una disciplina exclusivamente masculina (Slobodian 2021). La herpetología no es una excepción, aunque ahora hay muchas más investigadoras en este campo que hombres (Rock 2021). En general, la proporción de autoras es menor, al menos en la población que publica activamente (Salerno et al. 2019). En el caso de las descripciones de nuevas especies de *Pristimantis*, el número de mujeres que trabajan activamente en este grupo es escandalosamente bajo. En consecuencia, las mujeres aparecen escasamente como investigadoras principales, posición que se considera el indicador más importante acerca de las funciones que asumen en el proceso de descripción. De un total de 66 autoras, sólo seis han participado como autoras principales. Es necesario reflexionar sobre este patrón, ya que es coherente con otros estudios sobre la infrarrepresentación de las mujeres en la ciencia (West et al. 2013, Fox et al. 2018, Salerno et al. 2019). Si se mantienen las tendencias actuales de participación femenina en la descripción de especies de *Pristimantis* durante los próximos 25 años, se alcanzaría una proporción del 0,5. Esto significaría que en este período la proporción de mujeres que participan en las descripciones de *Pristimantis* podría ser potencialmente igual a la de los hombres, por supuesto, sin considerar la proporción de autores principales. Ecuador, al ser el país con más investigadores, también tiene el mayor número de mujeres investigadoras; sin embargo, muchas de ellas son tesistas de investigadores principales con mayor trayectoria, por lo que su incursión en el estudio de la taxonomía del grupo es temporal. En este artículo animamos a la inclusión activa de jóvenes investigadoras interesadas en el escasamente poblado campo de la Taxonomía, promoviendo el avance de la diversidad de *Pristimantis* (Guerra-García et al. 2008). Asimismo, animamos encarecidamente a los investigadores masculinos a que abran las puertas de sus laboratorios a las mujeres, donde sus funciones no se limitan a las de ayudantes de campo o secciones específicas de las descripciones, sino también como líderes que asumen posiciones críticas en determinados trabajos taxonómicos. La mejor manera de evidenciar esto en el futuro será a través de la proporción de mujeres en puestos de autoría senior (Salerno et al. 2019).

Revistas revisadas por pares

Las revistas con mayor número de contribuciones de descripciones de ranas pluviales son Zootaxa y Revista de la Academia Colombiana de Ciencias, Exactas Físicas y Naturales. Esto pone de manifiesto dos cuestiones críticas: La primera es el problema de la taxonomía actual (es decir, la subestimación del área al ser considerada una ciencia básica), y la segunda es cómo lograr que otras revistas escalen a mejores posiciones si son locales, gratuitas y de acceso abierto. Esta pregunta no tiene una respuesta sencilla ya que el escalamiento de estas revistas dependerá totalmente de métricas como el factor de impacto (FI), cuyo efecto repercute en el ciclo interminable de no citar a las revistas que no tienen un FI medianamente alto. Publicar artículos puramente taxonómicos es cada vez más complejo, tanto por la disminución del número de investigadores interesados en el campo como por el número de revistas interesadas en este tema (Wägele 2011). En 2020, Zootaxa fue excluida del Journal Citation Report (JCR) de 2019 (publicación de 2020) por Clarivate Analytics debido al uso excesivo de autocitas. Tras una petición que incluía más de 3900 firmas de biólogos investigadores, Clarivate Analytics revocó la decisión (Parise Pinto et al. 2021). Esta incomprensión y falta de conocimiento sobre el funcionamiento de la taxonomía hace pensar en la dificultad de publicar los descubrimientos de nuevas especies y en qué revistas pueden ser elegidas para tal tarea. Zootaxa muestra su importancia no sólo porque ha incluido descripciones de más del 25% de la biodiversidad mundial conocida (Parise Pinto et al. 2021), sino que en el caso de *Pristimantis* es la principal revista en la que se han publicado descripciones.

Tipo de descripción

Es importante señalar que la gran mayoría de las descripciones de nuevas especies se han basado en datos morfológicos, y que durante los últimos 20 años la revolución molecular ha transformado el proceso de descripción, incluyendo secuencias de ADN que permiten el posicionamiento filogenético de los nuevos taxones. El continuo aumento de las técnicas moleculares disponibles ha hecho que los costes para aplicarlas sean cada vez más bajos y, por tanto, más accesibles. Por ello, la inclusión de la secuenciación se ha convertido en una herramienta habitual para comprender mejor las relaciones ancestrales-descendientes de los organismos vivos (Malakhov 2013). Sin embargo, hay que tener en cuenta que en la descripción de nuevas especies, la secuenciación no debe sustituir una evaluación taxonómica detallada con caracteres

morfológicos que permitan diferenciar claramente una especie de otra (Guerra-García et al. 2008). Posicionar la Taxonomía como una ciencia necesaria y emergente para descubrir y describir la biodiversidad, imprescindible por derecho propio. Esto es evidente en el caso de *Pristimantis*, tal y como se apoya en esta revisión, dada la tendencia al aumento del número de especies descritas y las descripciones de especies altamente crípticas son cada vez más frecuentes (Padial y De la Riva 2009, Páez y Ron 2019, Ortega-Andrade 2015).

Categorías de amenazas globales

Por último, en cuanto al estado de conservación de *Pristimantis*, destacamos que una proporción representativa de las especies descritas hasta la fecha (es decir, el 36,8 %) se encuentra en categorías de incertidumbre como No evaluada y Datos insuficientes. Este dato refleja la necesidad de una evaluación global sobre las especies de este género, debido a su elevado endemismo y riqueza de especies. Por otra parte, también pone de manifiesto el ritmo creciente de descripciones de especies del género. Las evaluaciones del estado de conservación son realizadas generalmente por la UICN mundial cada cuatro o cinco años (UICN 2022), período en el que es probable que haya entre 10 y 15 nuevas especies de *Pristimantis* por año. Por lo tanto, las evaluaciones de la lista roja mundial siempre irán un paso por detrás de la evaluación de los criterios de amenaza. Una alternativa para abordar esta cuestión podría ser a través de las evaluaciones de las listas rojas locales y regionales, que se actualizarían más fácilmente y podrían incluir otras variables para la categorización (Ortega-Andrade et al. 2021). Además, muchas de las nuevas especies de *Pristimantis* tienen áreas de distribución restringidas con poblaciones que se enfrentan a diversas amenazas (por ejemplo, Brito-Zapata et al. 2021), lo que implica un mayor grado de vulnerabilidad. Sin embargo, si no se realizan evaluaciones globales para muchas de estas especies, las prioridades de conservación del género siguen siendo subestimadas. En este trabajo incluimos las categorías propuestas por la lista roja global de la UICN (UICN 2022), ya que las evaluaciones locales y regionales de cada país no están actualizadas y homogeneizadas y por lo tanto no pueden ser comparables. Ecuador ha realizado el esfuerzo más reciente para una categorización más holística y actualizada, estableciendo el 50% de las especies de *Pristimantis* ecuatorianas bajo algún grado de amenaza (Ortega-Andrade et al. 2021). Esta estimación parece más alta que el 33,5% reportado por la UICN, probablemente debido a la proporción de especies no evaluadas o con información insuficiente. Todos

estos aspectos refuerzan la importancia de seguir invirtiendo no sólo en el avance de la investigación taxonómica del género, sino también en estrategias de conservación articuladas entre los países de la región.

Agradecimientos

Estamos muy agradecidos con Gorki Ríos-Alvear, David Brito-Zapata y Maria Antonia Izurieta por su ayuda en el ingreso de información en la base de datos de descripciones de *Pristimantis*. Esta investigación fue apoyada por la Universidad San Francisco de Quito USFQ a través de Becas COCIBA (proyecto HUBI ID 12268, 17475) a CRP. Agradecemos mucho los comentarios de Edgar Lehr y Juan Manuel Guayasamin, que ayudaron a mejorar el manuscrito.

Referencias

Armesto LO, Señaris JC (2017) Anuros del norte de los andes: patrones de riqueza de especies y estado de conservación. Papéis Avulsos de Zoologia (São Paulo) 57. doi:10.11606/0031-1049.2017.57.39

Arroyo SB, Serrano-Cardozo VH, Ramírez-Pinilla MP (2008) Diet, microhabitat and time of activity in a *Pristimantis* (Anura, Strabomantidae) assemblage. Phyllomedusa: Journal of Herpetology 7. doi:10.11606/issn.2316-9079.v7i2p109-119

Boulenger GA (1882) Catalogue of the Batrachia Salientias. Taylor and Francis, London, pp.

Brito-Zapata D, Reyes-Puig C, Cisneros-Heredia D, Zumel D, Ron SR (2021) Description of a new minute frog of the genus *Pristimantis* (Anura: Strabomantidae) from Cordillera del Condor, Ecuador. Zootaxa 5072: 351-372. doi:10.11646/zootaxa.5072.4.3

Canedo C, Haddad CFB (2012) Phylogenetic relationships within anuran clade Terrarana, with emphasis on the placement of Brazilian Atlantic rainforest frogs genus Ischnocnema (Anura: Brachycephalidae). Molecular Phylogenetics and Evolution 65: 610-620. doi:10.1016/j.ympev.2012.07.016

Coloma LA, Guayasamin JM (2022) William E. Duellman (1930–2022). Phyllomedusa: Journal of Herpetology 21: 103–111

de Queiroz K (2005) A unified concept of species and its consequences for the future of taxonomy. Proceedings of the California Academy of Sciences 56 196–215

Duellman WE, Lehr E (2009) Terrestrial-breeding frogs (Strabomantidae) in Peru. Natur und Tier Verlag, pp.

Duellman WE, Pramuk JB (1999) Frogs of the genus *Eleutherodactylus* (Anura: Leptodactylidae) in the Andes of northern Peru. Scientific Papers Natural History Museum, University of Kansas 13: 1–78

Frost D (2022) Amphibian Species of the World: an Online Reference. Version 6.1. Electronic Database accessible at https://amphibiansoftheworld.amnh.org/index.php. [accessed January, 30th.2022]

Fox CW, Ritchey JP, Paine CET (2018) Patterns of authorship in ecology and evolution: First, last, and corresponding authorship vary with gender and geography. Ecology and Evolution 8: 11492-11507. doi:10.1002/ece3.4584

González-Durán GA, Targino M, Rada M, Grant T (2017) Phylogenetic relationships and morphology of the *Pristimantis* leptolophus species group (Amphibia: Anura: Brachycephaloidea), with the recognition of a new species group in *Pristimantis* Jiménez de la Espada, 1870. Zootaxa 4243. doi:10.11646/zootaxa.4243.1.2

Guayasamin JM, Hutter CR, Tapia EE, Culebras J, Peñafiel N, Pyron RA, Morochz C, Funk WC, Arteaga A (2017) Diversification of the rainfrog *Pristimantis ornatissimus* in the lowlands and Andean foothills of Ecuador. Plos One 12. doi:10.1371/journal.pone.0172615

Guerra García JM, Espinosa Torre F, García Gómez JC (2008) Trends in taxonomy today: an overview about the main topics in taxonomy. Zoológica baetica, 19: 15–49.

Hedges SB, Duellman WE, Heinicke MP (2008) New World direct-developing frogs (Anura: Terrarana): molecular phylogeny, classification, biogeography, and conservation. Zootaxa 1737: 1– 182

Heinicke MP, Duellman WE, Hedges SB (2007) Major Caribbean and Central American frog faunas originated by ancient oceanic dispersal. Proceedings of the National Academy of Sciences 104: 10092–10097. doi:10.1073/pnas.0611051104

Heinicke MP, Duellman WE, Trueb L, Means DB, Macculloch RD, Hedges SB (2009) A new frog family (Anura: Terrarana) from South America and an expanded directdeveloping clade revealed by molecular phylogeny. Zootaxa 2211: 1–35

Hutter CR, Guayasamin JM (2015) Cryptic diversity concealed in the Andean cloud forests: two new species of rainfrogs (*Pristimantis*) uncovered by molecular and bioacoustic data. Neotropical Biodiversity 1: 36–59. doi:10.1080/23766808.2015.1100376

Illinois Wesleyan Univerisity 2022 Edgar Lehr, Ph.D. https://www.iwu.edu/biology/faculty/lehr.html [accessed 3th June 2022]

IUCN (2022) The IUCN Red List of Threatened Species. https://www.iucnredlist.org [accessed Accessed on 14 June 2022].

INABIO (Instituto Nacional de Biodiversidad) 2022 Colecciones científicas: Herpetología. http://inabio.biodiversidad.gob.ec/colecciones_inabio/ [accessed 3th June 2022]

Jiménez de la Espada M (1870) Fauna neotropicalis species quaedam nondum cognitae. Jornal de Sciências, Mathemáticas, Physicas e Naturaes 3: 57–65

Kaiser H, Barrio-Amorós CL, Rivas GA, Steinlein C, Schmid M (2015) Five new species of *Pristimantis* (Anura: Strabomantidae) from the coastal cloud forest of the Península de Paria, Venezuela. Journal of Threatened Taxa 7: 7047-7088. doi:10.11609/JoTT.o4197.7047-88

Lehr E, Aguilar C, Siu-Ting K, Carlos Jordán J (2007b) Three New Species of *Pristimantis* (Anura: Leptodactylidae) from the Cordillera De Huancabamba in Northern Peru. Herpetologica 63: 519-536. doi:10.1655/0018-0831(2007)63[519:Tnsopa]2.0.Co;2

Lehr E (2007) New eleutherodactyline frogs (Leptodactylidae: *Pristimantis*, *Phrynopus*) from Peru. Bulletin of the Museum of Comparative Zoology 159: 145–178

Lynch JD (1968) Systematic status of some Andean leptodactylid frogs with a description of a new species of *Eleutherodactylus*. Herpetologica 24: 289–300

Lynch JD (1979) Leptodactylid frogs of the genus *Eleutherodactylus* from the Andes of southern Ecuador. Miscellaneous Publication Museum of Natural History, University of Kansas 66: 1–62

Lynch JD (1971) Evolutionary relationships, osteology, and zoogeography of leptodactyloid frogs. University of Kan sas Museum of Natural History, Miscellaneous Pulications 53: 1–2

Lynch JD (1998) New species of *Eleutherodactylus* from the Cordillera Occidental of western Colombia with a synopsis of the distributions of species in western

Colombia. Revista de la Academia Colombiana de Ciencias Exactas, Físicas y Naturales 22: 117–148

Lynch JD, Duellman WE (1980) The *Eleutherodactylus* of the Amazonian slopes of the Ecuadorian Andes (Anura: Leptodactylidae). Miscellaneous Publication Museum of Natural History, University of Kansas 69: 1–86

Lynch JD, Duellman WE (1997) Frogs of the genus *Eleutherodactylus* in western Ecuador, Systematics, ecology, and biogeography. . Special Publication Natural History Museum, University of Kansas 23 1–236

Lynch JD, Rueda-Almonacid JV (1998) Additional new species of frogs (genus *Eleutherodactylus*) from cloud forests of eastern Departamento de Caldas, Colombia. Revista de la Academia Colombiana de Ciencias Exactas, Físicas y Naturales 22 287–298

MUSM Museo de Historia Natural de la Universidad de San Marcos (2022) División de Herpetología https://museohn.unmsm.edu.pe/ [accessed 3th June 2022]

Mendoza ÁM, Ospina OE, Cárdenas-Henao H, García-R JC (2015) A likelihood inference of historical biogeography in the world's most diverse terrestrial vertebrate genus: Diversification of direct-developing frogs (Craugastoridae: *Pristimantis*) across the Neotropics. Molecular Phylogenetics and Evolution 85: 50-58. doi:10.1016/j.ympev.2015.02.001

Meza-Joya FL, Torres M (2016) Spatial diversity patterns of *Pristimantis* frogs in the Tropical Andes. Ecology and Evolution 6: 1901-1913. doi:10.1002/ece3.1968

Ortega-Andrade HM, Rojas-Soto OR, Valencia JH, Espinosa de los Monteros A, Morrone JJ, Ron SR, Cannatella DC (2015) Insights from Integrative Systematics Reveal Cryptic Diversity in *Pristimantis* Frogs (Anura: Craugastoridae) from the Upper Amazon Basin. Plos One 10. doi:10.1371/journal.pone.0143392

Ortega-Andrade HM, Rodes Blanco M, Cisneros-Heredia DF, Guerra Arévalo N, López de Vargas-Machuca KG, Sánchez-Nivicela JC, Armijos-Ojeda D, Cáceres Andrade JF, Reyes-Puig C, Quezada Riera AB, Székely P, Rojas Soto OR, Székely D, Guayasamin JM, Siavichay Pesántez FR, Amador L, Betancourt R, Ramírez-Jaramillo SM, Timbe-Borja B, Gómez Laporta M, Webster Bernal JF, Oyagata Cachimuel LA, Chávez Jácome D, Posse V, Valle-Piñuela C, Padilla Jiménez D, Reyes-Puig JP, Terán-Valdez A, Coloma LA, Pérez Lara MB, Carvajal-Endara S, Urgilés M, Yánez Muñoz MH (2021) Red List assessment of

amphibian species of Ecuador: A multidimensional approach for their conservation. Plos One 16. doi:10.1371/journal.pone.0251027

Páez NB, Ron SR (2019) Systematics of *Huicundomantis*, a new subgenus of *Pristimantis* (Anura, Strabomantidae) with extraordinary cryptic diversity and eleven new species. ZooKeys 868: 1-112. doi:10.3897/zookeys.868.26766

Padial JM, De La Riva I (2009) Integrative taxonomy reveals cryptic Amazonian species of *Pristimantis* (Anura: Strabomantidae). Zoological Journal of the Linnean Society 155: 97–122. doi:10.1111/j.1096-3642.2008.00424.x

Padial JM, Grant T, Frost DR (2014) Molecular systematics of terraranas (Anura: Brachycephaloidea) with an assessment of the effects of alignment and optimality criteria. Zootaxa 3825: 1– 132

Padial JM, Miralles A, De la Riva I, Vences M (2010) The integrative future of taxonomy. Frontiers in Zoology 7. doi:10.1186/1742-9994-7-16

Parise Pinto, ÂP, Mejdalani G, Mounce R, Silveira LF, Marinoni L, Rafael JA (2021). Are publications on zoological taxonomy under attack?. Royal Society Open Science, 8(2): 201617. doi:10.1098/rsos.201617

Peters JA (1955) Herpetological type localities in Ecuador Revista Ecuatoriana de Entomología y Parasitología 2: 335–352

Pinto-Sánchez NR, Ibáñez R, Madriñán S, Sanjur OI, Bermingham E, Crawford AJ (2012) The Great American Biotic Interchange in frogs: Multiple and early colonization of Central America by the South American genus *Pristimantis* (Anura: Craugastoridae). Molecular Phylogenetics and Evolution 62: 954-972. doi:10.1016/j.ympev.2011.11.022

Pröhl H, Ron SR, Ryan MJ (2010) Ecological and genetic divergence between two lineages of Middle American tungara frogs Physalaemus (=Engystomops) pustulosus. BMC Evolutionary Biology 10. doi:10.1186/1471-2148-10-146

Pyron AR, Wiens JJ (2011) A large-scale phylogeny of Amphibia including over 2800 species, and a revised classification of extant frogs, salamanders, and caecilians. Molecular Phylogenetics and Evolution 61: 543-583. doi:10.1016/j.ympev.2011.06.012

Ramírez-Castañeda V (2020) Disadvantages in preparing and publishing scientific papers caused by the dominance of the English language in science: The case of Colombian researchers in biological sciences. Plos One 15. doi:10.1371/journal.pone.0238372

Reyes-Puig C, Yánez-Muñoz MH, Ortega JA, Ron SR (2020) Relaciones filogenéticas del subgénero *Hypodictyon* (Anura: Strabomantidae: *Pristimantis*) con la descripción de tres especies nuevas de la región del Chocó. Revista mexicana de biodiversidad: e913013

Rock KN, Barnes IN, Deyski MS, Glynn KA, Milstead BN, Rottenborn ME, Andre NS, Dekhtyar A, Dekhtyar O, Taylor EN (2021) Quantifying the Gender Gap in Authorship in Herpetology. Herpetologica 77. doi:10.1655/0018-0831-77.1.1

Ron SR, Carrión J, Caminer MA, Sagredo Y, Navarrete MJ, Ortega JA, Varela-Jaramillo A, Maldonado-Castro GA, Terán C (2020) Three new species of frogs of the genus *Pristimantis* (Anura, Strabomantidae) with a redefinition of the *P. lacrimosus* species group. ZooKeys 993: 121–155. doi:10.3897/zookeys.993.53559

Ron S, Merino-Viteri A, Ortiz D (2022) Anfibios del Ecuador. Version 2022.0. Museo de Zoología. https://bioweb.bio/faunaweb/amphibiaweb [accessed January, 3th.2022]

Taucce PPG, Nascimento JS, Trevisan CC, Leite FSF, Santana DJ, Haddad CFB, Napoli MF (2020) A New Rupicolous Species of the *Pristimantis conspicillatus* Group (Anura: Brachycephaloidea: Craugastoridae) from Central Bahia, Brazil. Journal of Herpetology 54. doi:10.1670/19-114

Torres-Carvajal O, Pazmiño-Otamendi G, Ayala-Varela F, Salazar-Valenzuela, D (2022) Reptiles del Ecuador. https://bioweb.bio/faunaweb/reptiliaweb [accessed June, 3th]

R Core Team (2022) R: A language and environment for statistical computing. In: Computing RFfS (Ed), Vienna, Austria.

Salerno PE, Páez-Vacas M, Guayasamin JM, Stynoski JL (2019) Male principal investigators (almost) don't publish with women in ecology and zoology. Plos One 14. doi:10.1371/journal.pone.0218598

Slobodian V, Soares KDA, Falaschi RL, Prado LR, Camelier P, Guedes TB, Leal LC, Hsiou AS, Del-Rio G, Costa ER, Pereira KRC, D'Angiolella AB, de A. Sousa S, Diele-Viegas LM (2021) Why we shouldn't blame women for gender disparity in academia: perspectives of women in zoology. Zoologia 38: 1–9. doi:10.3897/zoologia.38.e61968

Wägele H, Klussmann-Kolb A, Kuhlmann M, Haszprunar G, Lindberg D, Koch A, Wägele JW (2011) The taxonomist-an endangered race. A practical proposal for its survival. Frontiers in zoology, 8(1): 1–7.

West JD, Jacquet J, King MM, Correll SJ, Bergstrom CT (2013) The Role of Gender in Scholarly Authorship. Plos One 8. doi:10.1371/journal.pone.0066212

Woolbright LL (1985) Patterns of nocturnal movement and calling by the tropical frog *Eleutherodactylus* coqui. Herpetologica: 1–9

Zumel D, Buckley D, Ron SR (2021) The *Pristimantis* trachyblepharis species group, a clade of miniaturized frogs: description of four new species and insights into the evolution of body size in the genus. Zoological Journal of the Linnean Society 195: 315-354. doi:10.1093/zoolinnean/zlab044

Yánez-Muñoz MH, Meza-Ramos PA, Cisneros-Heredia DF, Reyes-Puig JP (2010) Descripción de tres nuevas especies de ranas del género *Pristimantis* (Anura: Terrarana: Strabomantidae) de los bosques nublados del Distrito Metropolitano de Quito, Ecuador. ACI Avances en Ciencias e Ingenierías 2. doi:10.18272/aci.v2i3.40

Tablas

Tabla 1. Tasas de crecimiento anual en las descripciones de *Pristimantis*

Time series	Total	Ecuador	Colombia	Peru	Venezuela	Brazil	Panama	Bolivia
1958-1989	3.5	4.4	4.1	3.9	3.2	1.4	0	0
1990-2021	3.2	2.6	3.7	5.4	3.7	3.3	2.4	6.1
2010-2021	2.6	4.7	1.02	2.1	2.3	6.1	2.7	0
Total number of descriptions	591	212	168	107	56	16	9	5

Tabla 2. Los 100 primeros autores clasificados por el número de especies que han descrito. Se incluyen los autores con tres o más descripciones de especies. Los 20 primeros autores están en negrita.

**Autor**	**Autor (país)**	**No de especies descritas**	**Autor**	**Autor (país)**	**No de especies descritas**
**1**	**J. D. Lynch (USA)**	**195**	51	D. Szekely (Romania)	5
**2**	**W. E. Duellman (USA)**	**82**	52	D. Zumel (Ecuador)	5
**3**	**E. Lehr (Germany)**	**36**	53	E. A. de Oliveira (Brazil)	5
**4**	**M. H. Yánez-Muñoz (Ecuador)**	**36**	54	E. J. Hernandez-Ruz (Brazil)	5
**5**	**S. R. Ron (Ecuador)**	**30**	55	G. A. Rivas-Fuenmayor (Venezuela)	5
**6**	**P. M. Ruiz-Carranza (Colombia)**	**25**	56	M. B. Perez (Ecuador)	5
**7**	**G. A. Boulenger (Belgium, Great Britain)**	**23**	57	T. Barbour (USA)	5
**8**	**J. M. Guayasamin (Ecuador)**	**20**	58	W. C. H. Peters (USA)	5
**9**	**C. Reyes-Puig (Ecuador)**	**18**	59	L. R. Rodrigues (Brazil)	4
**10**	**C. L. Barrio-Amoros (Spain)**	**17**	60	C. J. Goin (USA)	4

**11**	**J. B. Pramuk (USA)**	**17**	61	D. Armijos-Ojeda (Ecaudor)	4
**12**	**J. V. Rueda-Almonacid (Colombia)**	**16**	62	D. B. Means (USA)	4
**13**	**J. P. Reyes-Puig (Ecuador)**	**15**	63	D. Buckley (Spain)	4
**14**	**M. C. Ardila-Robayo (Colombia)**	**15**	64	D. M. Cochran (USA)	4
**15**	**J. A. Rivero (Puerto Rico)**	**13**	65	D. Rödder (Germany)	4
**16**	**D. Batallas (Ecuador)**	**12**	66	E. R. Wild (USA)	4
**17**	**A. Catenazzi (USA)**	**11**	67	E.A. Pereira (Brazil)	4
**18**	**E. La Marca (Venezuela)**	**11**	68	J. C. Cusi (Peru)	4
**19**	**J. Brito-Molina (Ecuador)**	**11**	69	J. Culebras (Spain)	4
**20**	**N. B. Paez (Ecuador)**	**11**	70	J. G. Martinez (Colombia)	4
21	D. F. Cisneros-Heredia (Ecuador)	10	71	J. J. Mueses-Cisneros (Colombia)	4
22	J. C. Sánchez-Nivicela (Ecuador)	10	72	J. M. Padial (Spain)	4
23	S. B. Hedges (USA)	10	73	J. M. Savage (USA)	4
24	R. von May (USA)	9	74	K. L. A. Guimarães (Brazil)	4

25	M. Rivera-Correa (Colombia)	8	75	L. Alves da Silva (Brazil)	4
26	P. A. Burrowes (USA)	8	76	M. J. Navarrete **(Ecuador)**	4
27	A. F. Arteaga-Navarro (Ecuador)	7	77	M. Jimenez de la Espada (Spain)	4
28	C. Aguilar (Peru)	7	78	M. Penhacek (Brazil)	4
29	F. J. M. Rojas-Runjaic (Venezuela)	7	79	P. J. R. Kok (Belgium)	4
30	G. Flores (USA)	7	80	S. Duarte-Marín (Colombia)	4
31	H. M. Ortega-Andrade (Ecuador)	7	81	A. Almendariz (Ecuador)	3
32	J. H. Valencia (Ecuador)	7	82	A. Espinosa de los Monteros (Mexico)	3
33	J. Moravec (República Checa)	7	83	A. J. Crawford (USA)	3
34	P. J. Venegas (Peru)	7	84	A. M. Suarez-Mayorga (Colombia)	3
35	V. L. Urgiles (Ecuador)	7	85	A. Varela-Jaramillo (Ecuador)	3
36	C. W. Myers (USA)	6	86	C. F. Walker (USA)	3
37	H. Kaiser (USA)	6	87	C. Teran (Ecuador)	3

38	J. A. Ortega (Ecuador)	6	88	E. E. Infante-Rivero ()	3
39	J. C. Chaparro (Peru)	6	89	E. R. Dunn (USA)	3
40	J. J. Ospina-Sarria (Colombia)	6	90	F. H. Test (USA)	3
41	J. M. Daza (Colombia)	6	91	F. Werner (Austria)	3
42	M. A. Donnelly (USA)	6	92	G. A. González-Durán (Colombia)	3
43	M. Schmid (Germany)	6	93	G. A. Maldonado-Castro (Ecuador)	3
44	P. Szekely (Romania)	6	94	J. C. Jordan (Peru)	3
45	S. M. Ramirez-Jaramillo (Ecuador)	6	95	J. Carrion (Ecuador)	3
46	A. G. Ruthven (USA)	5	96	J. J. Morrone (Argentina-Mexico)	3
47	C. R. Hutter (USA)	5	97	J. Köhler (Germany)	3
48	C. Steinlein (Germany)	5	98	J. Lescure (France)	3
49	D. C. Cannatella (USA)	5	99	J. S. Eguiguren (Ecuador)	3
50	D. J. Santana (Germany)	5	100	K. Sui-Ting (Peru)	3

Tabla 3. Las lenguas más comunes en las que se han descrito nuevas especies de *Pristimantis*.

**Lenguaje**	**Número de descripciones**	**% Taxa**
Inglés	492	83.2
Español	73	12.4
Alemán	13	2.2
Portugués	4	0.7
Frances	4	0.7
Sueco	3	0.5
Italiano	1	0.2
Latin	1	0.2

Tabla 4. Las nacionalidades de los autores más comunes en las descripciones de *Pristimantis*.

**Lenguaje**	**Número de descripciones**	**% de autores**
Ecuatoriano	67	20.9
Americano-USA	60	18.8
Colombiano	49	15.3
Brasileño	30	9.4
Peruano	21	6.5
Alemán	18	5.6
Panemeño	7	2.2

Tabla 5. Las 21 autoras principales clasificadas por el número de especies que han descrito. * El autor principal se define como el primer autor o el autor correspondiente.

#	Autoras	Nacionalidad	Taxa	Autor Principal*
1	C. Reyes-Puig	Ecuatoriana	18	13
2	J. B. Pramuk	Americana-USA	17	17
3	M. C. Ardila-Robayo	Colombiana	15	-
4	N. B. Paez	Ecuatoriana	11	11
5	P. A. Burrowes	Americana-USA American	8	-
6	V. L. Urgiles	Ecuatoriana	7	5
7	J. A. Ortega	Ecuatoriana	6	-
8	M. A. Donnelly	Americana-USA	6	-
9	D. Szekely	Romanian	5	-
10	M. B. Perez	Ecuatoriana	5	-
11	M. J. Navarrete	Ecuatoriana	5	2
12	D. M. Cochran	Americana-USA	4	3
13	K. L. A. Guimarães	Brasileña	4	-
14	A. Almendariz	Ecuatoriana	3	-
15	A. M. Suarez-Mayorga	Colombiana	3	-
16	A. Varela-Jaramillo	Ecuatoriana	3	-
17	C. Teran	Ecuatoriana	3	-
18	G. A. Maldonado-Castro	Ecuatoriana	3	-
19	K. Sui-Ting	Peruana	3	-
20	P. Bejarano-Muñoz	Ecuatoriana	3	-
21	Y. Sagredo	Ecuatoriana	3	-

Figuras

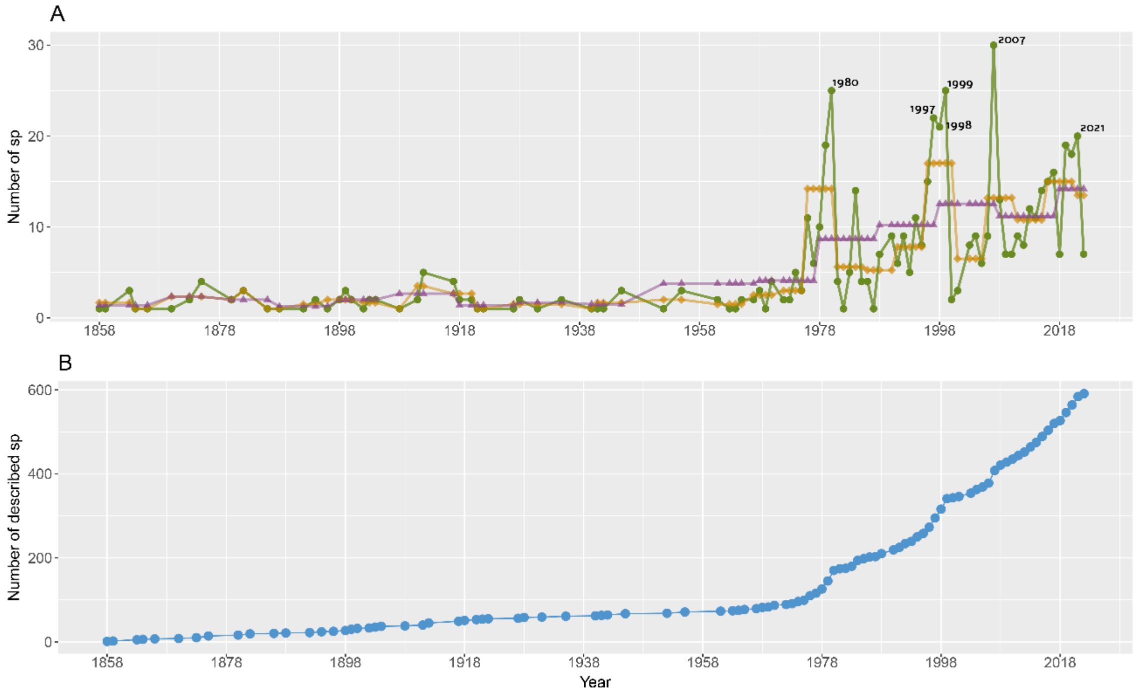

Figura 1. Descripciones de *Pristimantis* a lo largo del tiempo; A. Descripción de especies a lo largo de 164 años, los puntos y las líneas verdes representan nuevos taxones por año, los diamantes y las líneas marrones la media de cinco años, y los triángulos y las líneas moradas la media de diez años; B. Número acumulado de descripciones de especies.

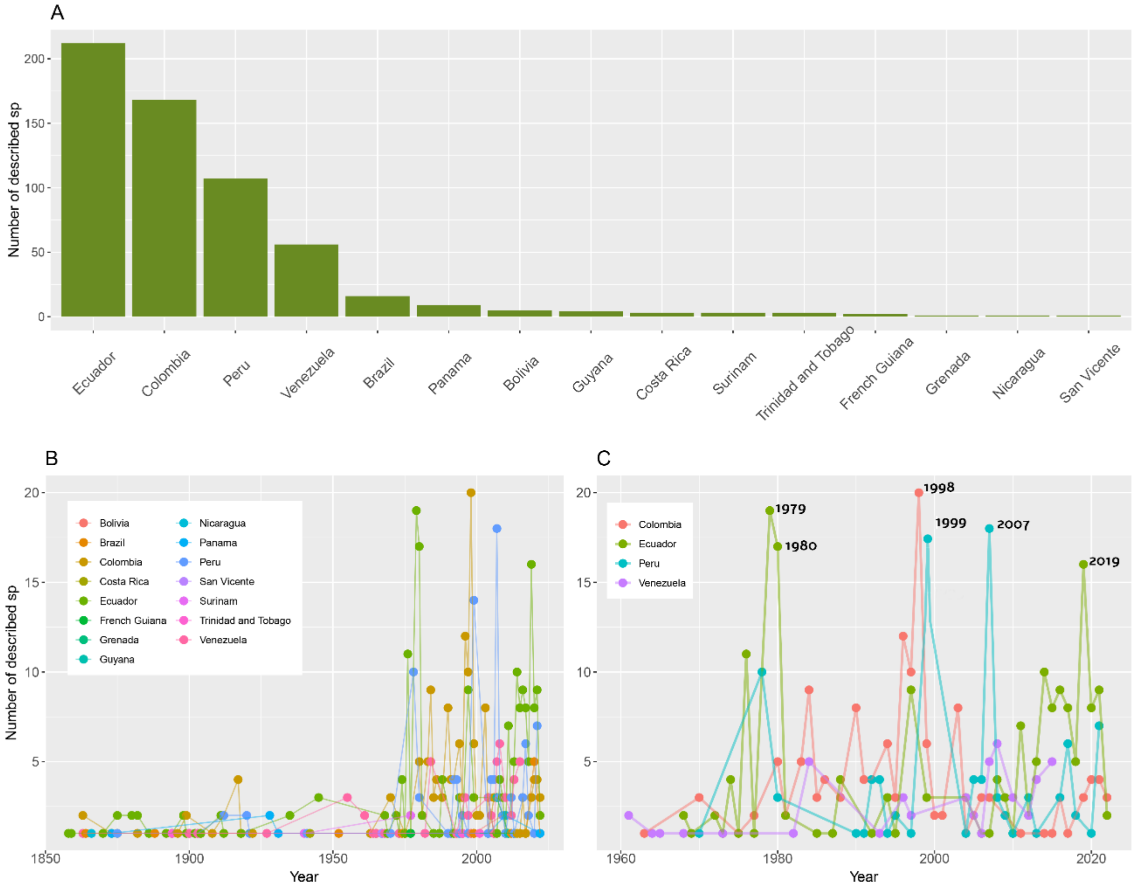

Figura 2. Descripciones de especies de *Pristimantis* en los distintos países; A. Número de descripciones de especies por país; B. Número de descripciones de especies anuales por país; C. Número de descripciones de especies anuales en los cuatro países con mayor riqueza de especies.

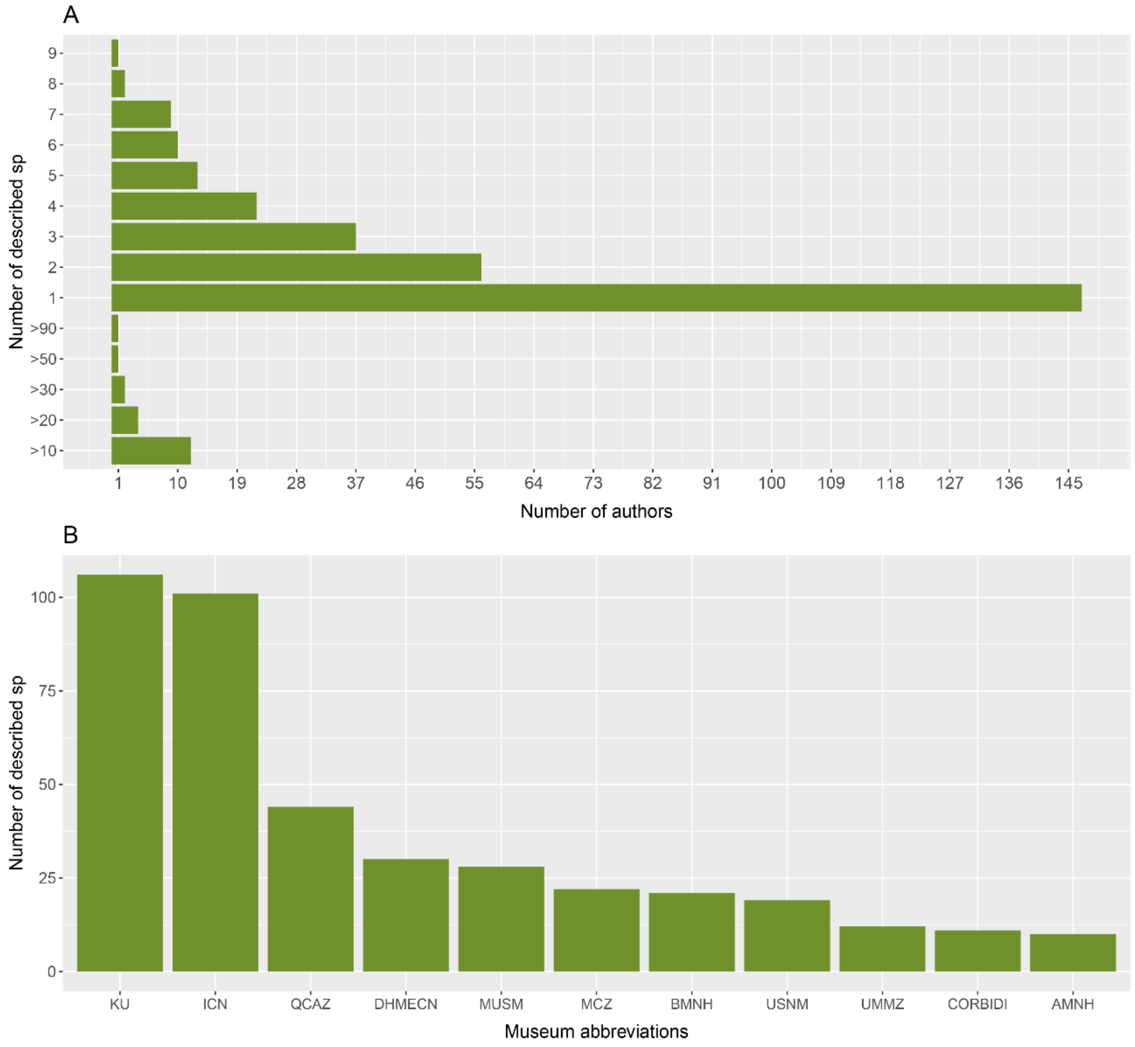

Figura 3. A. Número de especies descritas por número de autores; B. Número de holotipos de *Pristimantis* albergados en las principales colecciones científicas de historia natural (una lista ampliada se encuentra en el Material Suplementario 1)

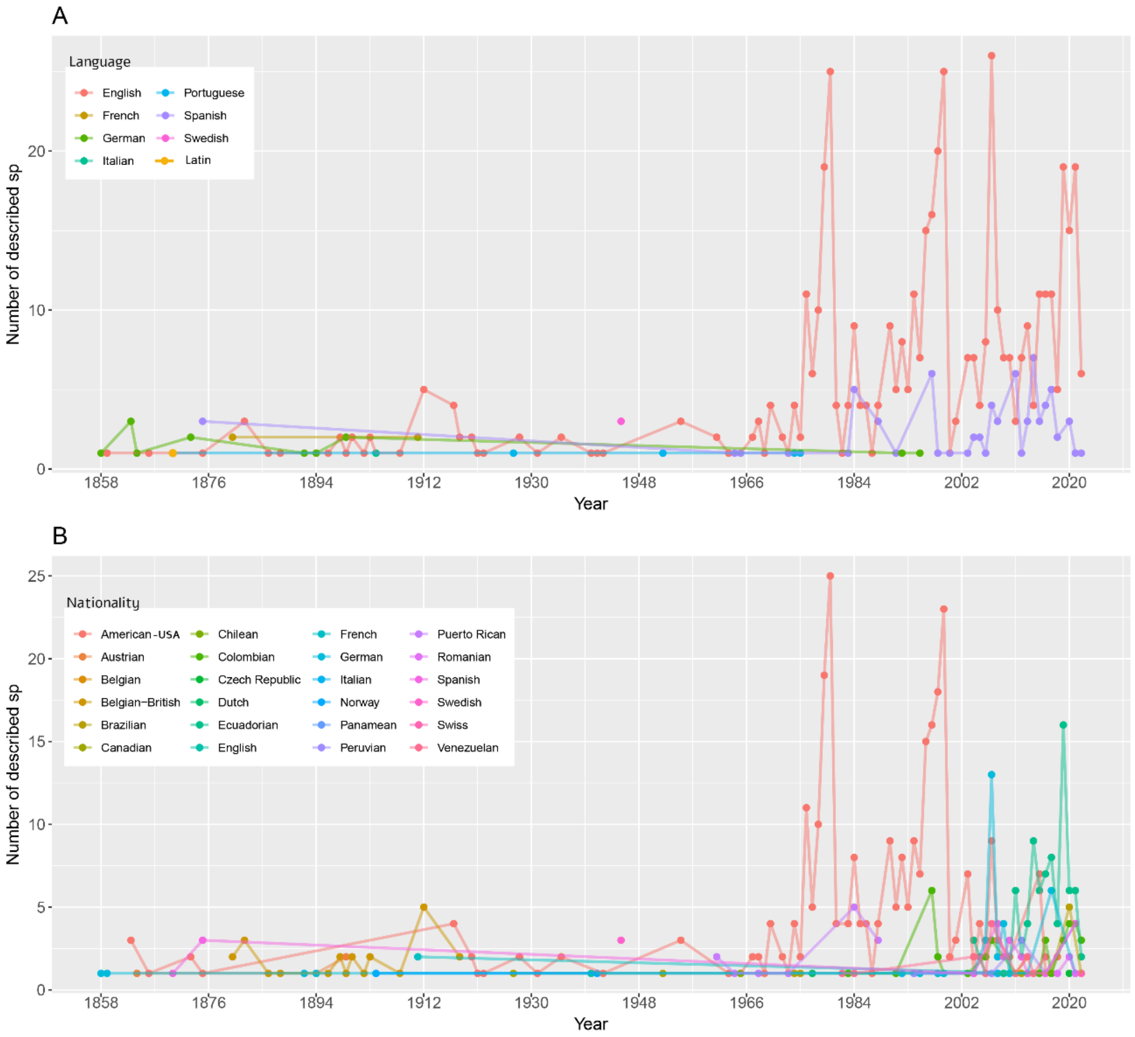

Figura 4. Idiomas y nacionalidades de los autores que han participado en las descripciones de las especies de *Pristimantis*; A. Principales idiomas utilizados en las descripciones por año; B. Principales nacionalidades de los autores que han participado en las descripciones por año.

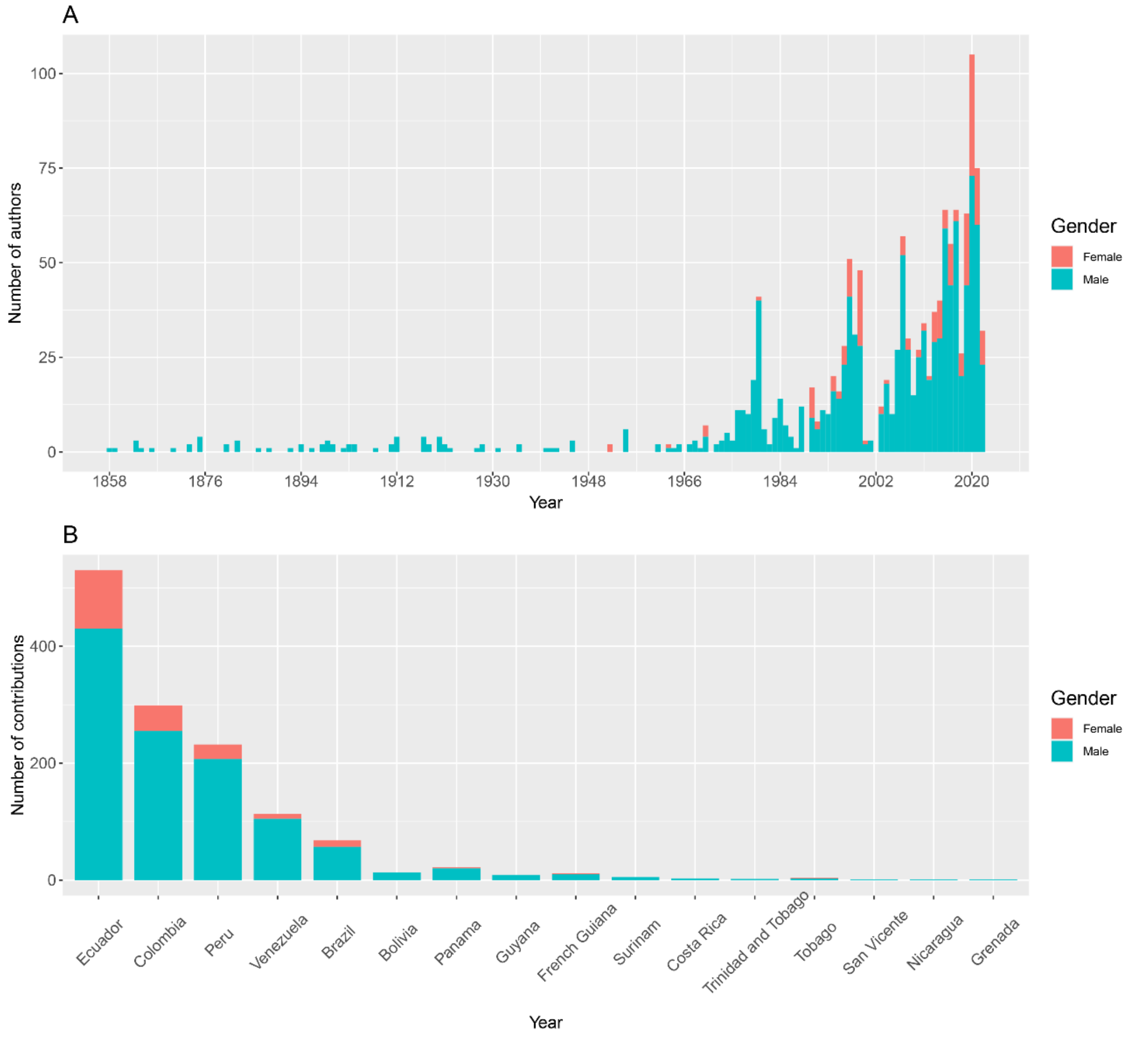

Figura 5. Contribuciones masculinas y femeninas a las descripciones de *Pristimantis*; A. Número de autores femeninos y masculinos que participaron en las descripciones por

año; B. Contribuciones de autores femeninos y masculinos a las descripciones por país.

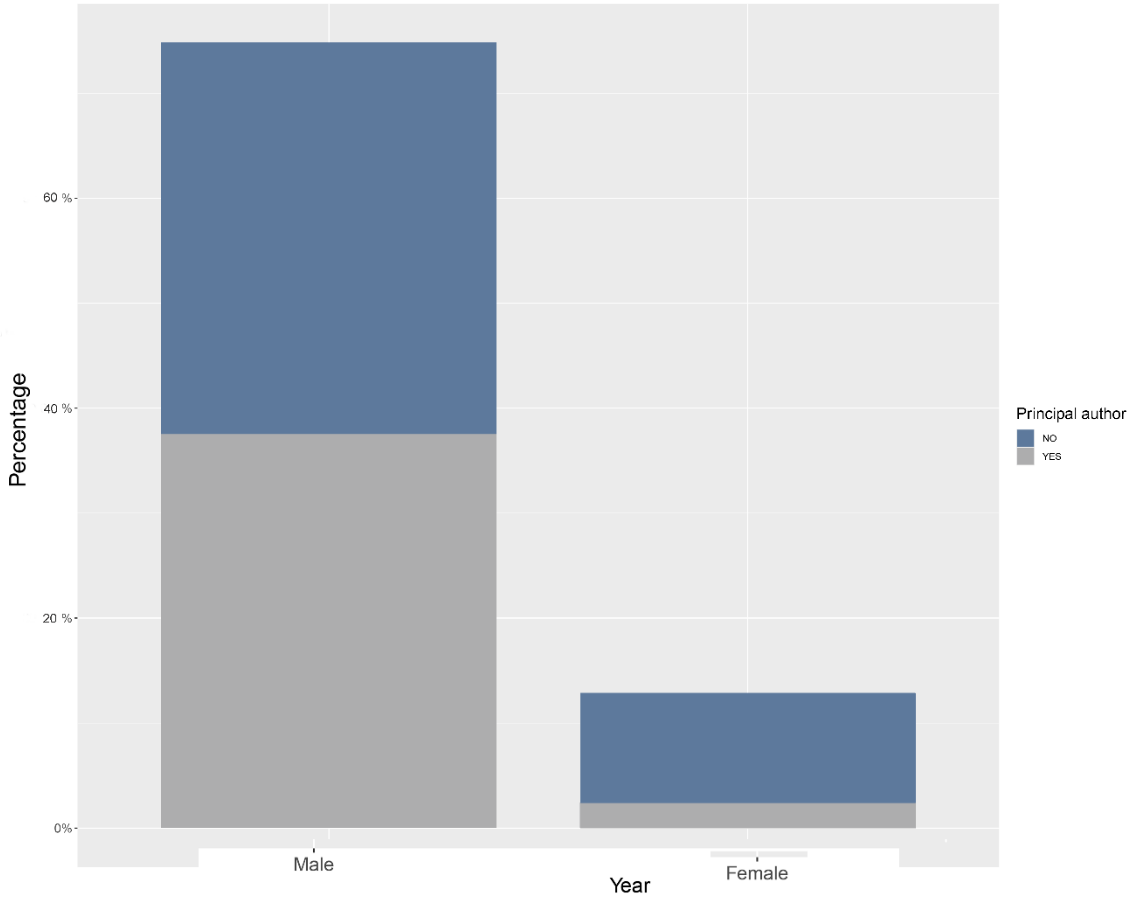

Figura 6. Contribuciones en las que los autores han sido autores principales * Definido como primer autor o autor correspondiente

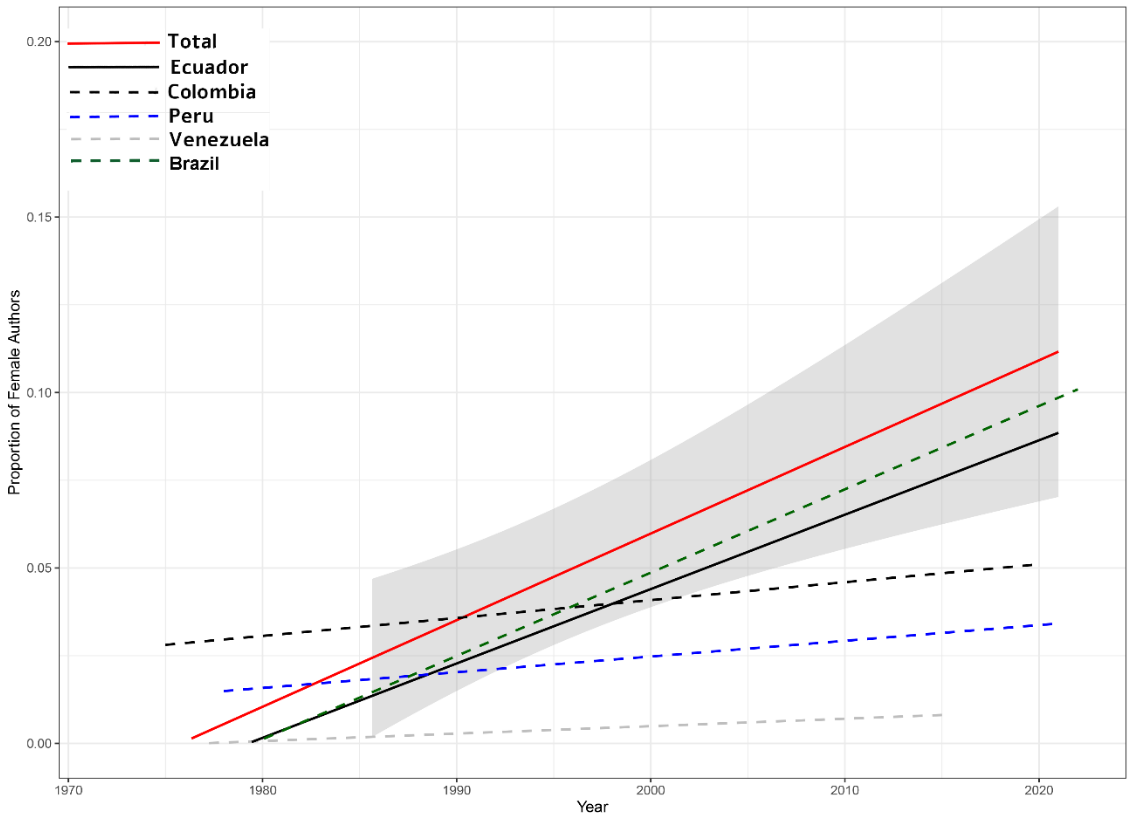

Figura 7. Cambio en la proporción de mujeres autoras a lo largo del tiempo. La sombra gris representa los intervalos de confianza del MLG de la proporción total de investigadoras (línea sólida roja). Las líneas continuas representan los MLG significativos.

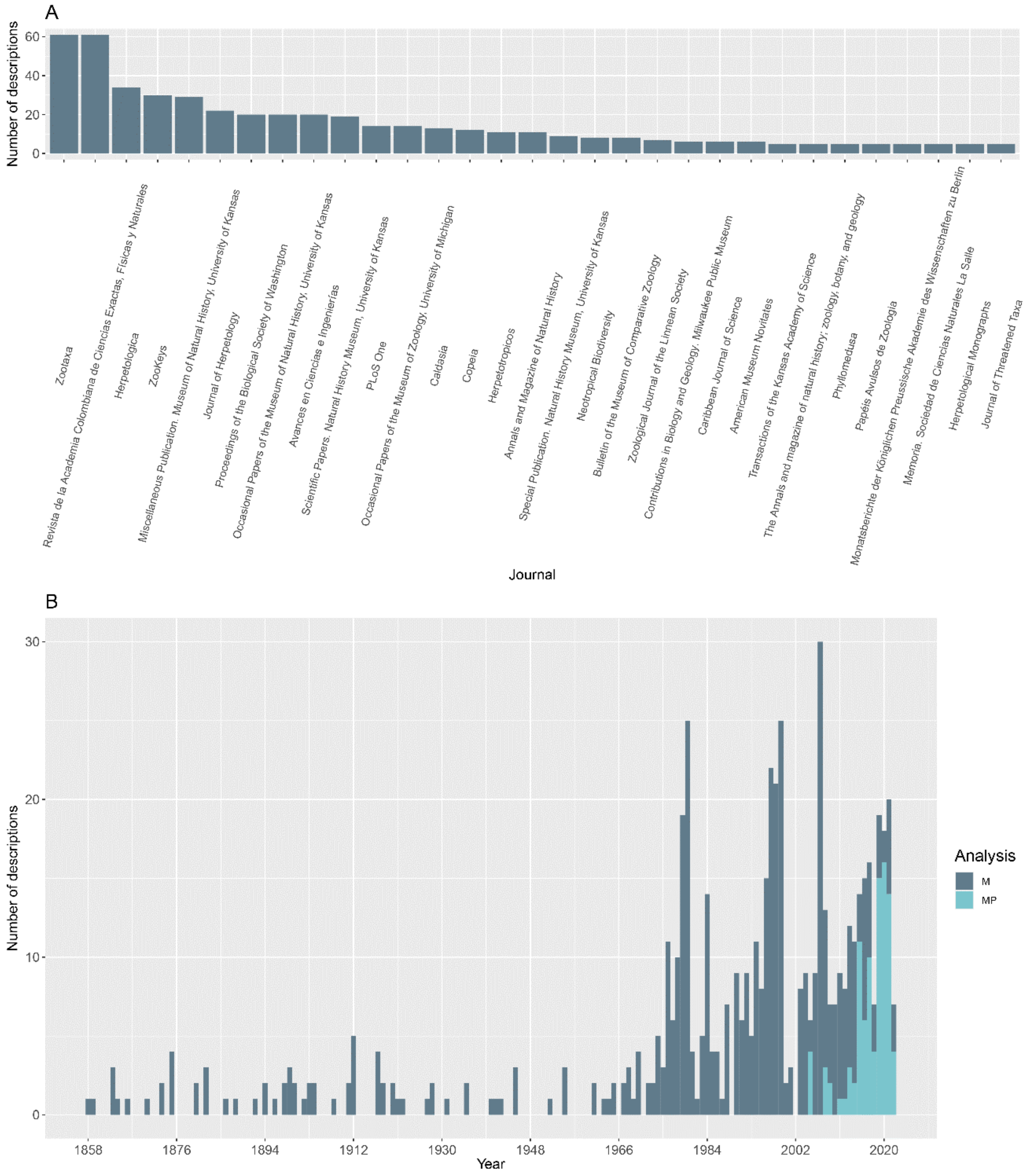

Figura 8. A. Principales revistas científicas donde se publican descripciones de nuevas especies de *Pristimantis*; B. Tipo de descripciones por año, M = Descripción morfológica, MP = Descripción morfológica y filogenética.

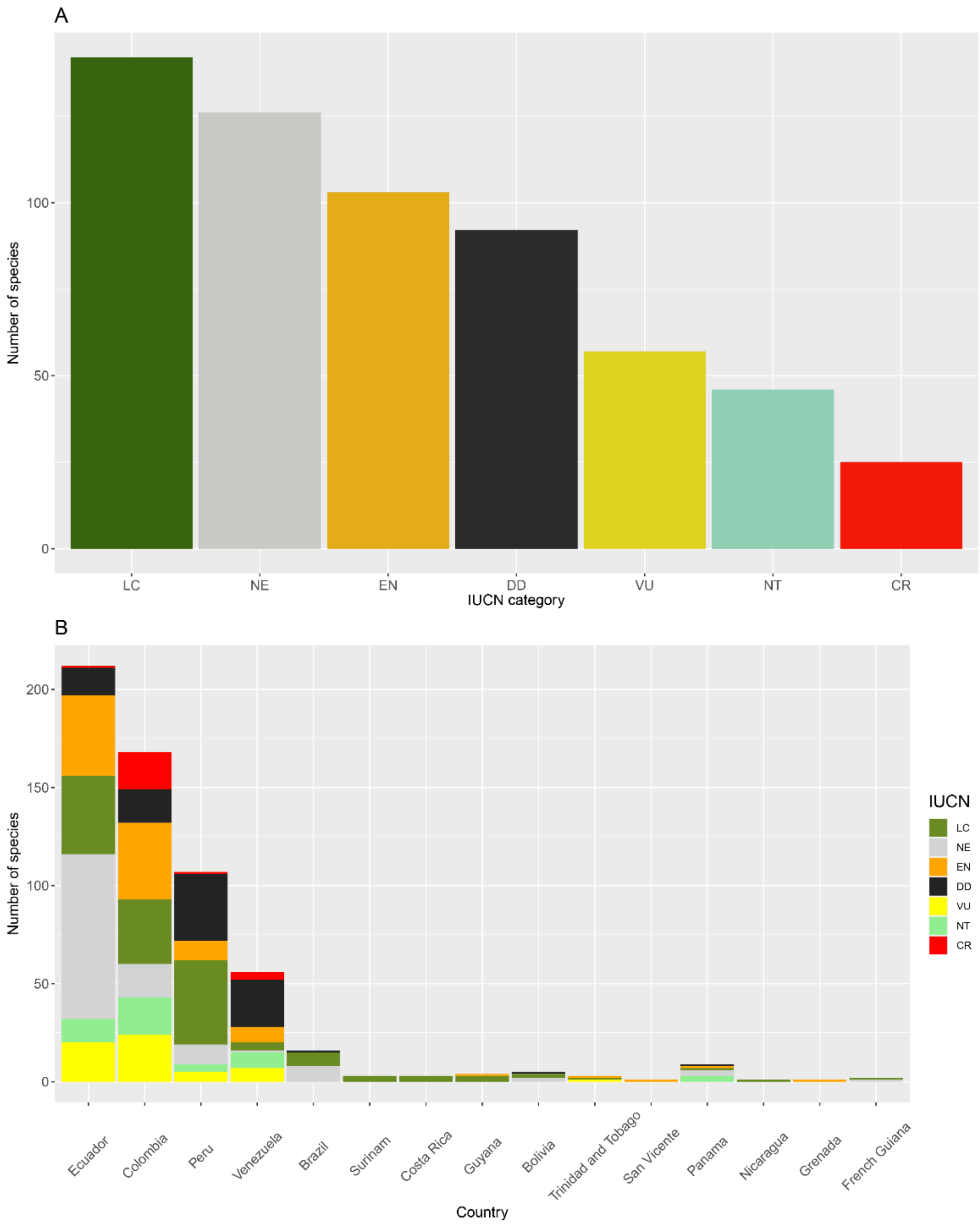

Figura 9. Categorías de estado de conservación establecidas por la Lista Roja de la UICN para las especies de *Pristimantis*; A. Número de especies por categoría. B. Número de especies por categoría y por país.
